# Analysing cluster randomised controlled trials using GLMM, GEE1, GEE2, and QIF: results from four case studies

**DOI:** 10.1186/s12874-023-02107-z

**Published:** 2023-12-13

**Authors:** Bright C. Offorha, Stephen J. Walters, Richard M. Jacques

**Affiliations:** Division of Population Health, School of Medicine & Population Health, University of Sheffield, Sheffield, UK

**Keywords:** Cluster randomised controlled trial, Statistical models, SAS, Intracluster correlation coefficient, Statistical methods

## Abstract

**Background:**

Using four case studies, we aim to provide practical guidance and recommendations for the analysis of cluster randomised controlled trials.

**Methods:**

Four modelling approaches (Generalized Linear Mixed Models with parameters estimated by maximum likelihood/restricted maximum likelihood; Generalized Linear Models with parameters estimated by Generalized Estimating Equations (1st order or second order) and Quadratic Inference Function, for analysing correlated individual participant level outcomes in cluster randomised controlled trials were identified after we reviewed the literature. We systematically searched the online bibliography databases of MEDLINE, EMBASE, PsycINFO (via OVID), CINAHL (via EBSCO), and SCOPUS. We identified the above-mentioned four statistical analytical approaches and applied them to four case studies of cluster randomised controlled trials with the number of clusters ranging from 10 to 100, and individual participants ranging from 748 to 9,207. Results were obtained for both continuous and binary outcomes using R and SAS statistical packages.

**Results:**

The intracluster correlation coefficient (ICC) estimates for the case studies were less than 0.05 and are consistent with the observed ICC values commonly reported in primary care and community-based cluster randomised controlled trials. In most cases, the four methods produced similar results. However, in a few analyses, quadratic inference function produced different results compared to the generalized linear mixed model, first-order generalized estimating equations, and second-order generalized estimating equations, especially in trials with small to moderate numbers of clusters.

**Conclusion:**

This paper demonstrates the analysis of cluster randomised controlled trials with four modelling approaches. The results obtained were similar in most cases, however, for trials with few clusters we do recommend that the quadratic inference function should be used with caution, and where possible a small sample correction should be used. The generalisability of our results is limited to studies with similar features to our case studies, for example, studies with a similar-sized ICC. It is important to conduct simulation studies to comprehensively evaluate the performance of the four modelling approaches.

**Supplementary Information:**

The online version contains supplementary material available at 10.1186/s12874-023-02107-z.

## Background

Randomisation is used in clinical trials to achieve balance between treatment arms in variations caused by both known and unknown prognostic factors, eliminate selection bias, and improve the external validity of the study. If done properly, it should minimise the effect of the prognostic factors so that researchers can controllably study the effect of the intervention(s) of interest [1]. Instead of randomising individuals to the treatment arms as done in individually randomised controlled trials (IRCTs), groups/clusters of individuals are randomised in cluster randomised controlled trials (CRCTs). In CRCT there are two levels; the distinctive cluster level and the individual level (with correlated outcomes) which are nested within the clusters. An appropriate statistical method for analysing CRCTs will be any method that considers this hierarchical nature of the CRCT design. Ignoring the correlated outcomes within a cluster and using standard statistical methods that treat the outcomes as being independent, might lead to underestimating the standard errors of the parameters and consequently obtaining narrower confidence intervals, false small P-values, and incorrectly overstating the effect of the intervention.

Some of the common issues in CRCT design and analysis are (a) Ignoring clustering [2], (b) inadequate handling of missing data [3], (c) and poor reporting of results [2, 4]. Newer analytical methods for handling clustering have been proposed in the literature of other study designs with clustered data, such as longitudinal study designs. Notable ones are targeted maximum likelihood estimation (TMLE) [5], quadratic inference function (QIF) [6], and alternating logistic regression (ALR) [7]. Furthermore, QIF is acclaimed to be a promising alternative to GEE1, especially when the correlation structure is misspecified [6, 8, 9], however, it is worth noting that these recent alternatives have not been comprehensively compared to the existing methods used in CRCTs like the GEE1, which might account for their slow uptake. This study aims to contribute to the literature (in the context of CRCTs) on the performance of the newer methods compared to the existing methods, to promote their use in CRCTs (if necessary).

This paper reviews and describes the selected statistical methods for analysing both continuous and binary outcomes in CRCTs. We focus on statistical methods for analysing individual-level outcomes which are correlated within clusters. The paper explores the performance of all the analytical methods given the settings of our case studies. The objectives of this study are to demonstrate the practical application of these selected modelling approaches for analysing CRCTs, to compare and discuss their methodological differences, and to make general comments based on our findings.

### Literature review

#### Search strategy

This review provides an overview of the appropriate and available statistical methods for analysing outcome data from CRCTs by mapping the evidence in the published literature on the development, refinement, and comparison of the statistical methods. This was a methodological review focusing on the appropriate, and available methods for analysing CRCTs with clustering in treatment arms. We reviewed the literature from 1^st^ January 2003 to 19^th^ December 2020. This was a year before the publication of the CONSORT statement 2004 extension for cluster randomised controlled trials.

We used a developed search strategy (see, Additional file 1) to search the online bibliography databases of MEDLINE, EMBASE, PsycINFO (via OVID); and CINAHL (via EBSCO), and SCOPUS. In addition to searching published literature databases, OpenGrey, web-of-science, and Scopus databases for conference proceedings were also searched to identify difficult-to-locate (grey) literature. A standardised pre-piloted data collection tool was used to extract information on the study and methodological characteristics from the included articles. One reviewer, BCO, carried out the search and extraction of the relevant information; two other independent reviewers, SJW and RMJ, supervised and validated the process. We discussed extensively to reach a consensus on issues presented during the review process.

### Literature search results

**Fig. 1:**
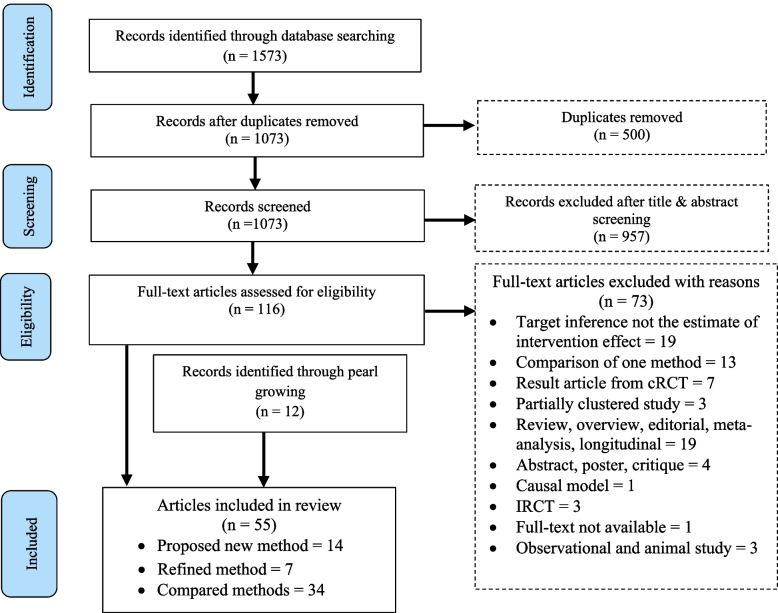
Flow chart of the search and selection process of the included articles

The literature search identified 1573 articles and after removing duplicates 1073 articles remained. After screening the title and abstract of each of the identified articles, 116 were shortlisted and 55 articles (including 12 from pearl growing) were finally chosen, while other 73 articles were excluded for various reasons (see, Fig. 1). These articles are methodological and application papers and are referenced throughout. The search and selection process of the included articles is presented in Fig. 1. Among the included 55 included articles; 34 (62%) compared already existing methods, 25% proposed new statistical methods, and 13% refined already existing ones. There was no clear pattern in the development, advancement, or comparison of statistical methods for analysing outcome data from CRCTs in the last two decades (see, Additional file 2).

The number of times each method was studied in the 55 articles and their references are summarised in Table S1 (see, Additional file 3). This review identified 27 unique statistical methods for analysing CRCTs which were studied 112 times in total. Regression models with parameters estimated by first-order generalized estimating equations (GEE1) was the most studied method (23/112, 21%) followed by maximum likelihood estimation (MLE) (16%). Among the newer methods, QIF was the most studied method (5%). Hence, four statistical regression models for the analysis of correlated individual participant-level outcomes in cluster randomised controlled trials were selected. They are:

- 1. Generalized Linear Mixed Models (GLMM) with parameters/coefficients estimated by Maximum likelihood (MLE) or restricted MLE denoted as GLMM henceforth.
- 2. Marginal Generalized Linear Models (mGLM) with parameters/coefficients estimated by 1st order Generalized Estimating Equations denoted as GEE1 henceforth.
- 3. Marginal Generalized Linear Models (mGLM) with parameters/coefficients estimated by 2nd-order Generalized Estimating Equations denoted as GEE2 henceforth.
- 4. Marginal Generalized Linear Models (mGLM) with parameters/coefficients estimated by Quadratic Inference Function denoted as QIF henceforth.

Specifically, GLMM and GEE1 were selected based on their popularity in the literature of CRCTs, they are the two most studied regression methods (see, Table S1), while GEE2 and QIF were selected based on findings that suggested them to be the two most promising improvements on the GEE1 [10–13]. GEE2 and QIF are not commonly used for analysing CRCTs, however, QIF has been extensively studied and applied in the context of longitudinal studies where outcomes measured repeatedly over time from a particular individual are likely to be correlated. For example, Odueyungbo et al., [9] and Song et al., [8] compared QIF to GEE1 using real-world data from longitudinal studies. Several other papers have compared QIF to GEE1 using both real-world and computer-simulated data, both in the context of longitudinal and CRCT designs [6, 14–17]. Similarly, several studies have compared GLMM to GEE1 to assess their relative performance [18–21]. To the best of our knowledge, no study has compared these four selected methods – GLMM, GEE1, GEE2, and QIF at the time of writing this report.

## Methods

### Notation

A boldface letter denotes either a vector or a matrix or as otherwise stated. The general notation is established as; let ${y}_{ij}$ denote an outcome for the $j$ ^th^ subject in the $i$^th^ cluster ($i=1,\dots ,N; j=1, \dots , {n}_{i})$; $N$ is the number of independent clusters in the study and ${n}_{i}$ denotes the different number of subjects in each cluster (i.e., the $i$ ^th^ cluster size), ${y}_{ij}$ has a corresponding set of $p$-dimensional vector covariates ${{\varvec{X}}}_{pij}^{T}= ({ x}_{1i},\cdots , {x}_{\mathrm{p}ij})$ where ${x}_{1i}$ denotes an indicator variable for the treatment group to which a cluster belongs $( {x}_{1i}=0$ indicates the control group and ${x}_{1i}=1$ the intervention group) and ${{\varvec{Y}}}_{i}= {({y}_{i1},\cdots , {y}_{{in}_{i}})}^{T}$ is a ${n}_{i}\times 1$ vector of the collection of the individual level outcomes for the $i$ ^th^ cluster. Also, ${{\varvec{\beta}}}_{p}= ({\beta }_{0},{\beta }_{1},\cdots , {\beta }_{p})$ is the unknown $p$-dimensional vector of regression parameters and ${{\varvec{\mu}}}_{i}= {({\mu }_{i1},\cdots , {\mu }_{i{n}_{i}})}^{T}$ is an ${n}_{i}\times 1$ vector of true means with ${\mu }_{ij}=E({y}_{ij }|{{\varvec{X}}}_{pij}^{T})$ being the conditional expectation for the $j$ ^th^ subject in the $i$ ^th^ cluster with covariates ${{\varvec{X}}}_{pij}^{T}$.

### Individual Level Analysis (ILA)

All the analytical methods considered in this study are based on individual-level analysis, meaning that outcomes from all the participating individual subjects in a trial are used as response values. This approach is further categorised according to how the regression model adjusts for clustering of the response values of subjects within a cluster. The different regression models and statistical methods used for estimating the regression coefficients in the models are explained in the subsequent subsections.

### Cluster-Specific Model (CSM)

The models classed under this category adjust for clustering by using the outcome of each of the subjects and conditionally relating it to the fixed effects and random effects components of the model. The parameter estimates of the fixed effects and random effects components are obtained simultaneously. The estimate of the intervention effect from this analytical approach is interpreted as what will happen to individuals in a cluster if they receive the intervention treatment compared to them receiving the control treatment. The linear mixed model (LMM) is a common example of this approach.

### Generalized Linear Mixed Model (GLMM) with coefficients estimated by MLE/REML

The GLMM is also called a random (or mixed) effects model and is the most used conditional/cluster-specific model for analysing CRCTs [2, 3]. The LMM, with a continuous outcome and identity link function is a special case of a GLMM. In a GLMM, a single model equation is specified to assess the impact of the fixed effects of some covariates of interest and the random effects of the randomly selected clusters on the outcome of interest. MLE is commonly used to estimate the parameters of the fixed effects and random effects components of a GLMM, simultaneously.

However, technically, the MLE algorithm estimates the fixed effects component initially (ignoring the random effects component), then plugs the estimates into the algorithm to estimate the random effects component. This process is repeated until optimal estimates are obtained. However, ignoring the random effects component in the first step causes the MLE to produce negatively biased variance components, because, it means ignoring the variations present in the estimates of the fixed effects, which could be substantial when the sample size is small [22–24]. Also, the MLE does not adjust for the degrees of freedom (DoF) lost in estimating the parameters of the fixed effects component [24]. Hence, the MLE is likely to produce SEs that are too small, resulting in smaller P-values, and inflated Type I error rates, especially when there are few clusters.

An alternative likelihood-based estimation method is the restricted maximum likelihood estimation (REML) which can be utilised to circumvent these problems. For large sample sizes, these problems are not noticeable, and the estimates from MLE and REML are approximately the same. However, for cRCTs with small samples, the problems are more pronounced [21, 23]. The REML first transforms the outcome data to remove the fixed effects, before estimating the random effects component. Then, it applies generalized least squares estimator to obtain the estimates of the fixed effects component within its algorithm. Put differently, REML obtains the estimates of the fixed effects and random effects components separately, starting with the random effects component [24]. To appropriately adjust for the loss in the DoF, we applied the Satterthwaite correction on the DoF, which resulted in adjusted P-values and CIs [21].

Let ${y}_{ij}$ denote a continuous outcome from a $j$ ^th^ individual in an $i$ ^th^ cluster. A specific example of the LMM called the random intercept LMM (because it adjusts for the random cluster effects using a random intercept term in the mixed model) is given as

1 $${y}_{ij}={\beta }_{0}{+{\beta }_{1}{x}_{1i}+\dots +\beta }_{p}{x}_{pij}+{\tau }_{i}+{\varepsilon }_{ij}, i=1,\dots ,N; j=1,\dots ,{n}_{i}, {\tau }_{i}\sim N\left(0,{\sigma }_{b}^{2}\right);{\varepsilon }_{ij}\sim N(0, {\sigma }_{w}^{2})$$

where ${\beta }_{1}$ is the intervention effect, ${x}_{1i}$ and ${x}_{pij}$ are the indicator and *p*^*th*^ variables respectively for the $j$ ^th^ individual in the $i$ ^th^ cluster, ${\tau }_{i}$ is the random effects term which causes variability in the cluster means and ${\varepsilon }_{ij}$ is the residual for each individual. When ${y}_{ij}$ is a non-Normally distributed outcome, such as a binary or count outcome, model Eq. (1) can be generalized. This explains the “generalized” in GLMM, the GLMM could be expressed as

2 $$\eta \left(E\left({y}_{ij}\right)\right)=\eta \left({\mu }_{ij}\right)={\beta }_{0}{+{\beta }_{1}{x}_{1i}+\dots +\beta }_{p}{x}_{pij}+{\tau }_{i}$$

where ${y}_{ij}$ is a non-normal outcome, $\eta (.)$ is a link function that linearly relates the expected response values to the fixed effects and the random effects components of the model. For example, if ${y}_{ij}\sim Bi\left(n,\mathrm{Pr}\left({y}_{ij}=1\right)\right)$ then Eq. (2) is specified using a logit link function as

3 $$logit \left(\mathrm{Pr}\left({y}_{ij}=1\right)\right)={\beta }_{0}{+{\beta }_{1}{x}_{1i}+\dots +\beta }_{p}{x}_{pij}+{\tau }_{i}$$

where $\mathrm{Pr}\left({y}_{ij}=1\right)$ is the probability of a success, that is, ${y}_{ij}=1$ and $logit \left(\mathrm{Pr}\left({y}_{ij}=1\right)\right)=\frac{\mathrm{Pr}\left({y}_{ij}=1\right)}{\left(1 -\mathrm{Pr}\left({y}_{ij}=1\right)\right)}$. MLE is a common choice for estimating the parameters of the GLMM. The general full likelihood of Eqs. (1), (2) and (3) is given as [25]

4 $$l\left({\varvec{\theta}},{\tau }_{i} ;{y}_{ij}\right)={\prod }_{ i=1}^{N}\int {\prod }_{j=1}^{{n}_{i}}\psi \left({\tau }_{i},{\varvec{\theta}}\right)g\left({\tau }_{i};{\sigma }_{b}^{2}\right)\partial {\tau }_{i}$$

where $l$(.) is the likelihood function for ${y}_{ij},\psi (.)$ is the probability function for ${y}_{ij}, {\tau }_{i}$ is often assumed to follow a Normal probability function $g$(.) and ${\varvec{\theta}}=( {\beta }_{0},{{\beta }_{1},\beta }_{p} )$. Maximum likelihood estimates are obtained by taking the first derivatives of the log of $l$(.) for each parameter, while the second derivative produces the standard errors. It is difficult to analytically obtain a closed-form solution for Eq. (4) due to the high dimension of the integral involved, a numerical likelihood approximation method is often used to circumvent this problem. We used the Adaptive Gauss-Hermite Quadrature (AGHQ) to perform the numerical approximation [26]. The GLMM models were implemented using the SAS 9.4 procedure; *PROC GLIMMIX*.

### Population Average Model (PAM)

The regression models under this class are appropriate for assessing the population average intervention effect. Here, inferences are made regarding the population of clusters rather than the individual subjects, and the target of the conclusions reached in the study is the population from where the clusters were drawn. Here, the intervention effect estimate is interpreted as the comparison of the average change in the population means between the intervention and control groups. PAMs are based on the marginal likelihoods of the correlated response values from the *i*^th^ cluster, ${{\varvec{Y}}}_{i}$, hence are considered to be semi-parametric models. The correlation of outcomes within clusters are accounted for using a separate working covariance matrix characterised by a working correlation matrix. In general, a PAM could be expressed as

5 $$\eta \left(E\left({{\varvec{Y}}}_{i}\right)\right)=\eta \left({{\varvec{\mu}}}_{i}\right)={{\varvec{X}}}_{pij}^{T} {{\varvec{\beta}}}_{p}$$

where $\upmu_{i}$ is the mean for the ith cluster. The marginal variance of a univariate response value ${y}_{ij}$ is often specified as $\phi \nu ({\mu }_{ij})$, where $\nu (.)$ is a known variance function and $\phi$ is a scale parameter that equals 1 for a binary outcome and ${\sigma }^{2}$ for a continuous outcome (and needs to be estimated). Equation (5) is similar to [2], but different in that corr $({\varepsilon }_{ij},{\varepsilon }_{{ij}{\prime}})\ne 0$ but rather corr $\left({\varepsilon }_{ij},{\varepsilon }_{{ij}{\prime}}\right)= \rho \left({x}_{ij},{x}_{i{j}{\prime}};{\varvec{P}}\right)\forall j\ne {j}{\prime}, {\varvec{P}}$ is the true correlation matrix to be approximated by a “working” correlation matrix, ${\varvec{R}}$, which is characterised by the intracluster correlation coefficient (ICC), $\rho$.

### The intracluster correlation coefficient

The ICC quantifies the correlation between the outcomes of any pair of subjects within a cluster. When the ICC is zero it indicates that any randomly paired outcome values from any randomly paired subjects in a cluster are independent, which gives rise to the “independence” working correlation structure. It is more common in cRCT to assume that the ICC is the same and nonzero across clusters which gives rise to the “exchangeable” working correlation structure. The independence and the exchangeable working correlation structures are the two most assumed in CRCTs. Common estimators of the ICC for continuous and binary outcomes are given as

6 $$\widehat{\rho }=\frac{{\upsigma }_{b}^{2}}{{\upsigma }_{b}^{2}+{\upsigma }_{w}^{2}} \mathrm{and} \widehat{\rho }=\frac{{\upsigma }_{b}^{2}}{{\upsigma }_{b}^{2}+\frac{{\pi }^{2}}{3}} \mathrm{respectively}$$

where ${\upsigma }_{b}^{2}$ is the intracluster variation, ${\upsigma }_{w}^{2}$ is individual subject variation and $\pi =3.141593$ [27]. These two parameters, ${\upsigma }_{b}^{2}$ and ${\upsigma }_{w}^{2}$, can be estimated using the extracts from the output of a one-way analysis of variance (ANOVA). According to Donner [28] the following equations hold true

7 $$\begin{aligned} {\widehat{\upsigma }}_{b}^{2}=\left(MSB-MSW\right)/\overline{n}\\ {\widehat{\upsigma } }_{w}^{2}=MSW \end{aligned}$$

where $MSB$ is the between-cluster mean squared error, $MSW$ is the within-cluster mean square error, both $MSB,$ and $MSW$ are the extracts from ANOVA, $\overline{n }$ is the average cluster size calculated with the formula below

8 $$\overline{n }=\frac{1}{N-1}\left(n-\frac{\sum_{i=1}^{N}{n}_{i}^{2}}{n}\right)$$

where *N* is the total number of clusters, n is the total sample size, and ${n}_{i}$ is the *i*^th^ cluster size. If Eq. (8) is substituted into Eq. (7) the ICC estimator becomes [29]

9 $$\widehat{\rho }=\frac{MSB-MSW}{MSB+\left(\overline{n }-1\right)MSW}$$

Obtaining either a positive or negative ICC estimate depends on which estimator is used, while the ICC estimator of Eq. (6) is positive definite because its components are variances, the other estimator, Eq. (9), can produce a negative ICC estimate because of the subtraction in its numerator, and this occurs when $MSB<MSW$.

### mGLM with coefficients estimated by GEE1

The first-order generalized estimating equations (GEE1) is the most common multilevel statistical method used for obtaining the parameter estimates of an mGLM (aka, PAM) specified in Eq. (5). The GEE1 estimator treats the correlations of outcomes within clusters as a nuisance, such that, it does not explicitly model the effect of the correlations. However, GEE1 accounts for the correlations using a separate “working” covariance matrix characterised by the working correlation matrix.

The GEE1 draws its strength from the linear exponential family distribution [30]. Liang and Zeger [31] proposed a class of estimating equations that uses a working correlation matrix (with fewer nuisance parameters) to obtain the parameter estimates of Eq. (5) given as

10 $${U}_{i}\left({\varvec{\beta}}\right)={\sum }_{ i=1}^{N}{\left(\frac{\partial {{\varvec{\mu}}}_{i}}{\partial{\varvec{\beta}}}\right)}^{T}{{\varvec{V}}}_{ i}^{-1}\left({{\varvec{Y}}}_{i} -{{\varvec{\mu}}}_{i}\left({\varvec{\beta}}\right)\right)=0$$

where ${{\varvec{V}}}_{i}$ is the ${n}_{i}\times {n}_{i}$ covariance matrix for ${{\varvec{Y}}}_{i}$ (i.e., ${{\varvec{V}}}_{i}=Cov{({\varvec{Y}}}_{i})$) specified by the working correlation matrix ${\varvec{R}}(\alpha )$ and defined as

11 $${{\varvec{V}}}_{i}=\phi {{\varvec{G}}}_{i}^\frac{1}{2}{ {\varvec{R}}}_{i}\left(\alpha \right){{\varvec{G}}}_{i}^\frac{1}{2}$$

where ${{\varvec{G}}}_{i}=diag\{\nu ({\mu }_{i1}), \cdots , \nu ({\mu }_{i{n}_{i}}) \}$ is a diagonal matrix with the diagonal elements $\nu ({\mu }_{ij})$ that is, the variance function for each response ${y}_{ij}$, and ${{\varvec{R}}}_{i}(\alpha )$ is an ${n}_{i}\times {n}_{i}$ working correlation matrix specified by the ICC, $\alpha$. Estimates from a GEE1 with an exchangeable correlation structure are equal to that of a random intercept model of Eq. (1) for linear models, but it is not necessarily the case for nonlinear models [32].The GEE1 estimator computes asymptotically consistent estimates $\widehat{{\varvec{\beta}}}$, regardless of the choice of ${{\varvec{R}}}_{i}(\alpha )$ but provided that the mean structure is correct. However, it may suffer some loss in efficiency if the choice of ${{\varvec{R}}}_{i}(\alpha )$ is not correct [6]. The parameter estimates $\widehat{{\varvec{\beta}}}$ are iteratively obtained by alternating between a modified Fisher scoring algorithm for ${\varvec{\beta}}$ and the moment estimation of $\alpha$ and $\phi$, and its residual ${N}^\frac{1}{2}(\widehat{{\varvec{\beta}}} -{\varvec{\beta}})$ is a multivariate Normally distributed residual with mean zero and a robust sandwich variance–covariance matrix ${{\varvec{\xi}}}_{i}$. The GEE1 models were fitted using the SAS 9.4 procedure, *PROC GENMOD*.

### mGLM with coefficients estimated by GEE2

This class of regression models attempts to leverage the major drawback of the GEE1 – possible loss in efficiency when the correlation structure is misspecified, especially when the correlation among outcomes is substantial [12, 13]. Statistical efficiency is a desirable property of a good estimator after unbiasedness has been established. Among all unbiased competing estimators, an efficient estimator is the one that produces the smallest standard error estimate, which is indicative of a lesser variability and a higher degree of precision.

The GEE2 model estimates the correlation parameter (i.e., the nuisance parameter in GEE1) and mean parameter simultaneously in its algorithm [11–13, 33, 34]. Hence, if modelling the correlation among subjects within a cluster is of primary interest, then GEE2 should be considered. For example, in a family study to assess the impact of the genetic relatedness of the family members on their alcohol dependence, GEE2 was highly recommended cause it may improve the efficiency of the mean parameters [13].

The models under the GEE2 analytical approach draw their strength from the quadratic exponential family distribution [30]. If the marginal density of ${{\varvec{Y}}}_{i}$ conditioned on the mean vector ${{\varvec{\mu}}}_{i}$ and the covariance matrix ${{\varvec{V}}}_{i}$, can be expressed as belonging to the quadratic exponential family distribution, then this allows for the mean and the covariance of ${{\varvec{Y}}}_{i}$ to be obtained simultaneously. Several GEE2 estimators have been proposed for estimating the mean and correlation parameters simultaneously [11, 12, 33, 34], however, Yan and Fine [13] used separate link functions to model the mean, the scale, and the correlation parameters and generated their corresponding sets of estimating equations to be solved simultaneously. This is known as the three-estimating Eqs. (3EE) GEE2, and it is applied in this paper.

To establish the model specification, let ${{\varvec{X}}}_{1i} , {{\varvec{X}}}_{2i}\ \mathrm{and}\ {{{\varvec{X}}}_{3i}}$ be the ${n}_{i}\times p, {n}_{i}\times r$ and $\frac{n(n+1)}{2}\times q$ design matrices for the mean, the scale, and the correlation parameters of the vector of outcomes ${{\varvec{Y}}}_{i}$, respectively. The specific link function for the mean, the scale, and correlation parameters to ${{\varvec{X}}}_{1i} , {{\varvec{X}}}_{2i}\ \mathrm{and}\ {{\varvec{X}}}_{3i}$, respectively, is given as

12 $$\begin{array}{c}{\eta }_{1}\left({{\varvec{\mu}}}_{i}\right)={{\varvec{X}}}_{1i}{\varvec{\beta}}\\ {\eta }_{2}\left({{\varvec{\phi}}}_{i}\right)={{\varvec{X}}}_{2i}\boldsymbol{\varphi }\\ {\eta }_{3}\left({{\varvec{\rho}}}_{i}\right)={{\varvec{X}}}_{3i}\boldsymbol{\alpha }\end{array}$$

where ${{\varvec{\mu}}}_{i}$ is a ${n}_{i}\times 1$ mean vector specified by ${\varvec{\beta}}$, ${{\varvec{\phi}}}_{i}$ is a ${n}_{i}\times 1$ scale vector specified by $\boldsymbol{\varphi }$ and ${{\varvec{\rho}}}_{i}$ is a $\frac{{n}_{i}({n}_{i}+1)}{2}\times 1$ pairwise correlation vector specified by $\boldsymbol{\alpha }$. The unified corresponding set of estimating equations for Eq. (12) to be solved simultaneously is given as

13 $${U}_{i}\left({\varvec{\beta}},\boldsymbol{\varphi },\boldsymbol{\alpha }\right) =\begin{array}{c}{\sum }_{ i=1}^{N}{\left(\frac{\partial {{\varvec{\mu}}}_{i}}{\partial{\varvec{\beta}}}\right)}^{T}{{\varvec{V}}}_{ i}^{-1}\left({{\varvec{Y}}}_{i} -{{\varvec{\mu}}}_{i}\left({\varvec{\beta}}\right)\right)=0 \\ {\sum }_{i=1}^{N}{\left(\frac{\partial {{\varvec{\phi}}}_{i}}{\partial \boldsymbol{\varphi }}\right)}^{T}{{\varvec{V}}}_{2i}^{-1}({{\varvec{Z}}}_{i} -{{\varvec{\phi}}}_{i}(\boldsymbol{\varphi }))=0\\ {\sum }_{i=1}^{N}{\left(\frac{\partial {{\varvec{\rho}}}_{i}}{\partial {\varvec{\upalpha}}}\right)}^{T}{{\varvec{V}}}_{3i}^{-1}\left({{\varvec{S}}}_{i} -{{\varvec{\rho}}}_{i}\left(\boldsymbol{\alpha }\right)\right)=0\end{array}$$

where ${{\varvec{Y}}}_{i}$ and ${{\varvec{V}}}_{1i}$ is as defined in the GEE1 mean model of Eqs. (10) and (11), ${{\varvec{Z}}}_{i}$ is the ${n}_{i}\times 1$ vector of the scales, ${{\varvec{S}}}_{i}$ is the $\frac{{n}_{i}({n}_{i}+1)}{2}\times 1$ vector of the pairwise correlations, ${{\varvec{V}}}_{1i}$ and ${{\varvec{V}}}_{2i}$ are the working covariance matrices of ${{\varvec{Z}}}_{i}$ and ${{\varvec{S}}}_{i}$ respectively.

The GEE2 (Eq. (13)) requires the specification of the first four central moments of the outcome vector (mean response, variance, skewness, kurtosis). Yan and Fine [13] suggested a way around it to avoid the problem of convergence and it is implemented using the *geese* [35] function in the R package *geepack* [36]. In general, the third and fourth moments can be specified as functions of the first and second moments, thereby avoiding the direct estimation of higher-order moments [12]. The GEE2 estimator consistently estimates the mean parameters ${\varvec{\beta}}$ regardless of whether the scale and correlation structures are wrong, the estimates for scales $\boldsymbol{\varphi }$ are consistent regardless of whether the working correlation is mis-specified, but provided that the mean and scale structures are correct.

The major merit of the 3EE GEE2 estimator is that it allows for separate covariates to be included in the mean, scale, and correlation models. This is important when investigating heterogeneous correlation across clusters or treatment arms, such as modelling multiple forms of clustering. Where each cluster or treatment arm presents a different degree of correlation ${\alpha }_{i}$ among subjects, possibly due to cluster sizes and covariates imbalance. Taking this heterogeneity into account may improve efficiency, instead of assuming a constant correlation across clusters or treatment arms [10]. The solutions of Eq. (13) are obtained iteratively by alternating between a modified Fisher scoring algorithm and the moment estimation method. The GEE2 models were fitted using the R’s *geese* function in the *geepack* package.

### mGLM with coefficients estimated by QIF

Similar to GEE2, the quadratic inference function (QIF) was proposed to circumvent a major issue with GEE1, that is, the loss in efficiency due to the misspecification of the correlation structure. But compared to GEE2, QIF does not require the specification of the third and fourth moments (as it imposes additional constraints). The QIF estimator avoids the direct use of the working correlation matrix in its algorithm. Instead, it uses a linear combination of basis matrices and some constants to replace the inverse of the working correlation matrix. Hence, the QIF is more robust to misspecification of the working correlation matrix compared to GEE1, providing better protection against incorrect correlation structure. With this, the QIF produces more efficient parameter estimates compared to GEE1 [6]. However, if the working correlation structure is not misspecified, the efficiency of the parameter estimates from GEE1 and QIF are equivalent [6, 8].

Let ${{\varvec{Y}}}_{i}, {{\varvec{X}}}_{i}, {{\varvec{\mu}}}_{i}$, and ${{\varvec{V}}}_{i}$ be the same as defined in Eqs. (10) and (11). In the QIF equation, the inverse of ${\varvec{R}}$ specified in Eqs. (10) and (11) is approximated using a linear combination of a set of several basis matrices ${{\varvec{R}}}_{h}^{-1}\approx {k}_{h}{{\varvec{M}}}_{h}+\dots +{k}_{m}{{\varvec{M}}}_{m}; \left(h=1,\dots ,m\right); {{\varvec{M}}}_{h}$ is the $h$th known basis matrix with its unknown coefficient/constant, ${{\varvec{k}}}_{h}$, that needs to be estimated. For the exchangeable and autoregressive working covariance matrix, $h=1$and 2 should suffice, respectively [6, 17]. Using this new information, we can rewrite the estimating Eq. (10) of the GEE1 as extended score equations given as

14 $${\overline{g} }_{N}\left({\varvec{\beta}}\right)=\frac{1}{N}{\sum }_{i=1}^{N}{g}_{i}\left({\varvec{\beta}}\right)\approx \frac{1}{N}\left(\begin{array}{c}{\sum }_{ i=1}^{N}{\left(\frac{\partial {{\varvec{\mu}}}_{i}}{\partial{\varvec{\beta}}}\right)}^{T}{{\varvec{G}}}_{i}^{-1}\left({{\varvec{Y}}}_{i} -{{\varvec{\mu}}}_{i}\left({\varvec{\beta}}\right)\right) \\ {\sum }_{ i=1}^{N}{\left(\frac{\partial {{\varvec{\mu}}}_{i}}{\partial{\varvec{\beta}}}\right)}^{T}{{\varvec{G}}}_{i}^{-{}^{1}\!\left/ \!{}_{2}\right.}{{\varvec{M}}}_{1}{{\varvec{G}}}_{i}^{-{}^{1}\!\left/ \!{}_{2}\right.}\left({{\varvec{Y}}}_{i} -{{\varvec{\mu}}}_{i}\left({\varvec{\beta}}\right)\right) \\ \vdots \\ {\sum }_{ i=1}^{N}{\left(\frac{\partial {{\varvec{\mu}}}_{i}}{\partial{\varvec{\beta}}}\right)}^{T}{{\varvec{G}}}_{i}^{-{}^{1}\!\left/ \!{}_{2}\right.}{{\varvec{M}}}_{m}{{\varvec{G}}}_{i}^{-{}^{1}\!\left/ \!{}_{2}\right.}\left({{\varvec{Y}}}_{i} -{{\varvec{\mu}}}_{i}\left({\varvec{\beta}}\right)\right) \end{array}\right)$$

where ${g}_{i}({\varvec{\beta}})$ is the score vector of each cluster, the constants ${{\varvec{k}}}_{m}$ are considered a nuisance and are not included. The QIF estimator uses the generalized method of moments (GMM) [37] to optimally combine the multiple estimating equations in [13]. Hence, the estimate $\widehat{{\varvec{\beta}}}$ is obtained by minimising the weighted length of ${\overline{{\varvec{g}}} }_{N}$ using the GMM, which could be express as

15 $$\widehat{{\varvec{\beta}}}=arg {min}_{{\varvec{\beta}}} {\overline{{\varvec{g}}} }_{N}^{T}{{\varvec{\Sigma}}}_{N}^{-1} {\overline{{\varvec{g}}} }_{N}$$

where $arg {min}_{{\varvec{\beta}}}$ is the argument of the minimum of ${\varvec{\beta}}$ that minimises ${\overline{{\varvec{g}}} }_{N}^{T}{{\varvec{\Sigma}}}_{N}^{-1} {\overline{{\varvec{g}}} }_{N}$. As expected, the true covariance matrix ${{\varvec{\Sigma}}}_{N}$ is replaced by the estimated covariance matrix ${{\varvec{C}}}_{N}$ in Eq. (15), with its inverse ${{\varvec{C}}}_{N}^{-1}$ representing a weighting function. Thus, the QIF estimator becomes

16 $${\widehat{Q}}_{N}\left({\varvec{\beta}}\right)={\overline{{\varvec{g}}} }_{N}^{T}{\mathbf{C}}_{N}^{-1} {\overline{{\varvec{g}}} }_{N}$$

where ${{\varvec{C}}}_{N}=\left(1/{N}^{2}\right){\sum }_{i}^{N}{g}_{i}\left({\varvec{\beta}}\right){g}_{i}^{T}\left({\varvec{\beta}}\right), {{\varvec{C}}}_{N}^{-1}$ is the main reason behind QIF’s efficiency advantage, because it weights the information each $i$ ^th^ cluster contributes to the estimating equation, clusters with large variation are given less weight than the ones with small variation. The estimates $\widehat{{\varvec{\beta}}}$ are obtained iteratively using the Newton–Raphson algorithm [6] to evaluate Eq. (16). The QIF models were fitted using the SAS 9.4 macro: *qif.*

### Comparison between the methods

Table 1 compares the methodological properties of the four modelling approaches, and some of these properties are discussed below. For ILA there are situations where the parameter estimates from CSM and PAM are equivalent in interpretation. A random intercept LMM typifying a CSM is equivalent to a PAM with an exchangeable working correlation structure and collapsible link function, however, both methods produce inconsistent estimates (i.e., biased estimates) when the cluster sizes are informative [32, 38, 39]. Theoretically, the random intercept LMM and PAMs with an exchangeable working correlation structure produce different parameter estimates in the case of noncollapsible link functions, and also if the cluster sizes are informative.

**Table 1:** Similarities and differences in the methodological properties of the four selected statistical models for analysing CRCTs

S/NO	Feature	GLMM	GEE1	GEE2	QIF
1	**Adjustment for clustering**	Clustering is accounted for via a random effects term with its coefficient and that of fixed effects term estimated simultaneously using a single mean model equation [25]	The structure of clustering is described using a separate working covariance matrix (characterised by the working correlation matrix) which is specified separately from the mean model equation [40]	A separate set of estimating equations and link functions are used to model the mean and correlation parameters, thereby explicitly explaining the source of the cluster-level variations [13]	Avoids the direct use of the correlation parameters in its algorithm, instead, it uses a linear combination of the product of basis matrices and some constants [6]
2	**Assumption on the distribution of the cluster-level random effects**	Most times in GLMM it is assumed that the cluster-level random effects follow a parametric distribution, and Normal distribution is a common choice	As a semi-parametric method, it does not assume any distribution for the cluster-level random effects	Same as GEE1	Same as GEE1
3	**Multiple forms of clustering**	Accommodates multiple forms of correlation to be investigated by incorporating them as random effects in the mean model	Allows multiple forms of correlation but through a complex procedure of including higher forms of clustering as fixed effects in the mean model	Same as GEE1	Same as GEE1
4	**Assumption of missing data mechanism required to obtain consistent parameter estimates**	Missing completely at random and missing at random	Missing completely at random [40]	Same as GEE1	Same as GEE1
5	**Heterogenous correlation**	Flexible in modelling complex correlation structures using multiple random effects variables	Not flexible in modelling data with complex correlation structure	More flexible than GEE1 by using a separate equation, link function, and covariates for the correlation parameter	Same as GEE1
6	**Improvement in efficiency (i.e., the treatment effect estimate with a smaller SE)**	Gain in efficiency by including random effects components in the mean model to account for correlation among outcomes in a cluster, especially when the correlation is large	Gain in efficiency by using a "working covariance matrix" which accounts for the effect of the correlation among outcomes in a cluster, however, it treats the correlation as a nuisance	More gain in efficiency compared to GEE1 by explicitly modelling the effect of the correlation among outcomes with a separate equation that allows covariates adjustment. This provides some protection against misspecification of the correlation structure	Firstly, it uses a different strategy that protects against the misspecification of the correlation structure. Secondly, it weights the information contributed by each cluster using an empirical weighting matrix, clusters with large variation are given less weight and vice versa. It is acclaimed that these two features increase its gain in efficiency compared to the GEE1
7	**Moment specification**	First and second-order moments are to be specified	Same as GLMM	The first four order moments^1^, but the third and fourth can be specified as a function of the first two moments since a working correlation is being used	Same as GLMM
8	**Approximation technique**	Laplace/Adaptive Gauss-Hermite Quadrature^2^	Modified Fisher scoring algorithm	Alternate between the Modified Fisher scoring algorithm and the method of the moment	Newton–Raphson algorithm
9	**Goodness of fit**	All the model selection criteria that are based on maximum likelihood theory are applicable, such as the LRT, AIC, and BIC	Uses a modification to the AIC based on a quasi-likelihood theory known as QIC (and QICu^3^) for model and working correlation selections	Same as GEE1	Provides an objective function that follows a chi-square distribution (which is analogue to the likelihood ratio test)
10	**Availability in selected statistical software, function(package)**	*R* = glmer(lme4) and SAS = glimmix(proc)	*R* = glmgee(geepack) and SAS = genmod(proc)	*R* = geese(geepack) only	*R* = qif(qif) and SAS = qif(macro)

*GLMM* Generalized linear mixed model, *GEE* Generalized estimating equations, *QIF* Quadratic inference function, *LRT* Likelihood ratio test, *AIC* Akaike information criteria, *BIC* Bayesian information criteria, *QIC* Quasi-likelihood independence criterion

1. The first four order moments of the outcome of interest are the mean, variance, skewness, and kurtosis

2. Adaptive Gauss-Hermite Quadrature equals the Laplace approximation when the quadrature point/node is 1. Other techniques do exist

3. QICu is a variant of QIC that allows for the correlation in the data but is not adequate for selecting a working correlation structure [41]

In terms of efficiency (concerning the size of the SE of the estimated treatment effect), the GEE1 considers the correlation among outcomes within clusters, this improves its efficiency (see, Table 1, row 6). However, GEE1 produces a consistent intervention effect estimate (and its SE) if the mean model is correct and outcome data are missing completely at random regardless whether the correlation structure is misspecified [31]. However, GEE1 suffers some loss in efficiency if the working correlation structure is not close to the true correlation structure, especially when the true correlation is large and/or the sample size is small. When the sample size is small (which is a recipe for imbalance) the robust SE estimator of GEE1 does not provide full protection over incorrect working correlation structure, causing GEE1 to have reduced efficiency in regards to the size of the SE of the estimated intervention effect [23, 40, 42].

This disadvantage of the GEE1 is the reason why GEE2 and QIF were developed to improve GEE1’s efficiency. GEE2 achieves this by explicitly modelling the mean and correlation parameters simultaneously, using separate sets of estimating equations. Also, if mean and correlation are of interest, GEE2 is more likely to produce efficient inferences for the mean and correlation parameters than GEE1, especially if the correlation within clusters is substantial and the sample size is small [10–13]. QIF is another alternative to GEE1 that uses a different strategy to estimate the working correlation parameter, thereby minimising the impact of its misspecification. Studies have proved this advantage of the QIF in the context of a longitudinal study [6, 8, 9]. Their results showed that QIF is more efficient than GEE1 when the true correlation is large and misspecified. Several authors have shown that this claim might not necessarily hold when there are few clusters and/or there is cluster and covariate imbalance between treatment arms [15–17].

The MLE as an estimator of GLMM is known to be consistent and efficient when the distributional assumptions made are correct. One such assumption is that the random cluster effects are Normally distributed. Previous studies had overstated the impact of misspecifying the distribution of the random effects on MLE [43, 44]. However, a recent study has shown that the MLE is quite robust to the impact of misspecifying the distribution of the random effects in most situations considered previously [45], even when the cluster size is informative [46].

The goodness-of-fit of a statistical model is a crucial part of building an optimal regression model for practical uses. Appropriate goodness-of-fit methods for CSMs have been extensively studied in the literature whereas goodness-of-fit methods for PAMs are few. The early goodness-of-fit methods for GEE-based models involve partitioning the covariates space into separate groups and then calculating their score statistics which are approximately Chi-square distributed [47, 48]. This strategy is an extension to that of Tsiatis [49] and Hosmer and Lemeshow [50] for uncorrelated outcomes. This strategy was found to produce different results in different statistical software because the partitioning is subjective to the software used [51], and this problem may likely extend to population average models for analysing correlated outcomes [41].

Pan (2001) [41] proposed a goodness-of-fit method for PAMs that mimics Akaike’s Information Criterion (AIC) known as the Quasi-likelihood information criterion (QIC). While AIC is based on maximum likelihood, QIC is based on quasi-likelihood under an independence working correlation structure in GEE1. The results of the simulation study conducted in the paper showed that the AIC was more efficient than the proposed QIC, however, the performance of the QIC was remarkable. The author did not clearly state if this criterion applies to GEE2 but noted that using the GEE2 approach to estimate the scale parameter included in their criterion is difficult. A goodness-of-fit method exists for GEE2 in McCullagh and Nelder (1989) [52]. To the best of our knowledge, the method is not available in standard statistical packages at the time of authoring this current paper.

Pan (2002) [53] further proposed two other tests for a logistic population average model; the Pearson chi-square G and the unweighted sum of squares U tests which are based on the Normal distribution with means and variances (using unstructured working correlation). When analysing a correlated binary outcome if the model has at least one continuous covariate, it becomes difficult to apply goodness-of-tests that are based on Chi-square distribution, because the partitioning of the continuous covariate would result in a situation where the total number of the distinct groups is bigger than the sample size. Hence, the Pan (2002) developed these two tests (Pearson chi-square G and the unweighted sum of squares U) to circumvent this problem.

QIF’s goodness-of-fit method is based on an objective function that is approximately chi-square distributed with appropriate DoF. It shares similar asymptotic properties to that of the likelihood ratio test, which is negative twice the log-likelihood [$-2\times (\mathrm{log}(l\left(.\right))$] [6]. This is one of the advantages QIF has over GEE1 [6, 8, 9]. The QIF’s objective function can be constructed from models with a working correlation structure different from the independence, unlike the GEE1’s QIC which is only based on an independence working correlation structure [41].

## Description of the four CRCT datasets

### PoNDER trial [54]

The PoNDER CRCT aimed to assess the effect of two psychologically informed interventions by health visitors on postnatal depression in postnatal women who have recently given birth. One hundred and one general practices (clusters) in the Trent region of England were included in the trial. The general practices were randomised in a 2:1 ratio to the Intervention group (*n* = 63 clusters) or the control group (*n* = 38 clusters). Health visitors in the intervention clusters were trained to identify depressive symptoms at six to eight weeks postnatally using the Edinburgh postnatal depression scale (EPDS) and were also trained in providing psychologically informed sessions based on cognitive behavioural or person-centred principles for an hour a week for eight weeks. Health visitors in the control group provided usual care.

The primary outcome was the score on the EPDS at six months follow-up. The EPDS consists of 10 questions and generates a score on a 0 to 30 scale with higher scores indicating a great risk of depression. For the PoNDER trial, this outcome was dichotomised into a binary outcome of EPDS score < 12 vs $\ge$ 12 with women with a score of 12 or more classified as “at risk” of postnatal depression. One hundred (*n* = 63 intervention, *n* = 37 control) clusters and *n* = 2659 new mothers (1745 Intervention: 913 Control) provided valid primary outcome data at 6 months. Also, one of the secondary outcomes in the PoNDER trial “the mean EPDS score at six months” was used as a continuous outcome in this study. In the original study, both outcomes were analysed using GEE1 and an exchangeable correlation structure with robust standard errors. The descriptive statistics of the trial size are presented in Table 2 below.

**Table 2:** A summary of the sample size of the four CRCTs analysed in this study

Trial	No. of clusters	No. of clusters missing	No. of subject	Missing n (%)	Average cluster size	(Min, Max) cluster size	Median cluster size
PoNDER	101	1	2659	35 (1)	27	(1, 101)	21
Informed Choice	10	0	1547	108 (7)	155	(74, 308)	145
Bridging the Age Gap	43	0	748	36 (5)	18	(1, 73)	16
NOSH	92	0	9207	0 (0)	100	(12, 333)	75

### Informed choice trial [55]

This study was aimed at investigating the impact of a set of 10 pairs of evidence-based leaflets – The Midwives’ Information and Resource Service (MIDIRS) and NHS Centre for Reviews and Dissemination informed choice leaflets through a survey. The study was designed to cover 8 of the 10 MIDIRS decision points in everyday maternity care. Conducted in 12 large maternity units in Wales, the maternity units were grouped into 10 clusters. Pairs of clusters were randomly assigned to the intervention arm and control arm based on their annual numbers of deliveries to achieve balance, and undertook an unmatched analysis.

The primary objective was to improve the management of women during pregnancy and childbirth, by assessing the effect of an intervention that promotes informed choice. The primary binary outcome was the change in the proportion of women who reported exercising informed choice (yes or no). For illustration, one of the secondary outcomes "the average of the women's levels of knowledge” on the 10 topics covered in the survey was used as a continuous outcome in this current study. Knowledge of the topics was assessed on a 1 (poor) to 10 (good) scale. Two samples of different women were surveyed: the antenatal and postnatal samples. The antenatal sample is made up of all women who reached 28 weeks’ gestation within six weeks and were receiving antenatal care in any setting. The questionnaire used for the cohort covered three decision points that the women may have encountered. The postnatal sample was made up of all women who delivered live babies during a six-week period.

A questionnaire that covered the remaining five decision points was used to survey the women postnatally. The postnatal sample had a total of 3,288 women, who were cross-sectionally surveyed before (*n* = 1,741) and after the intervention was administered (*n* = 1,547). However, to demonstrate the fitting of the statistical methods in this study only the follow-up (i.e., after the intervention) postnatal sample was used and reported. Only women who delivered in all settings and above the age of 16 years were included. Random effects models (i.e., GLMM) were used to analyse the outcomes in the original study. A summary of the trial size is presented in Table 2.

### Bridging the age gap trial [56]

Bridging the Age Gap CRCT investigated the effects of two decision support interventions (DESIs) to support treatment choices in older women (aged $\ge$ 70 years) with operable breast cancer [56]. Forty-six breast cancer units (clusters) in England and Wales were included in the trial. The breast cancer units were randomised to have access to the DESI (Intervention group n = 21 clusters) or to continue with usual care (Control group *n* = 25 clusters). The DESI comprised an online algorithm, booklet, and brief decision aid to inform choices between surgery plus adjuvant endocrine therapy versus primary endocrine therapy, and adjuvant chemotherapy versus no chemotherapy.

The primary outcome was the global health status/quality of life (QoL) score (questions 29 and 30) on the cancer-specific patient-reported outcome of the European Organisation for the Research and Treatment of Cancer (EORTC) QoL questionnaire (QLQ)-C30 at 6 months post-baseline. The EORTC QLC-C30 global health status/QoL scale is scored on a 0 to 100 scale with a higher score representing a better QoL. Forty-three clusters (*n* = 19 intervention, *n* = 24 control), and *n* = 748 patients (359 Intervention: 389 Control) provided valid primary outcome data at 6 months.

The primary endpoint was a continuous outcome “Global health status quality of life score” measured 6 months after diagnosis and was analysed using GEE1 with sandwich (robust) standard errors and an exchangeable working correlation matrix. The total number of participants included in the trial is 748 distributed across 43 clusters and the cluster size ranged from size 1 to 73. A summary of the trial size is provided in Table 2.

### The Nourishing Start for Health (NOSH) trial [57]

The NOSH CRCT assessed the effect of an area-level financial incentive (shopping vouchers) on breastfeeding among new mothers (and their baby(ies)) in areas with low breastfeeding prevalence [57]. Ninety-two electoral ward areas (clusters) in England were included in the trial with baseline breastfeeding prevalence at 6 to 8 weeks postnatally of less than 40%. The areas were randomised to the financial incentive plus usual care (*n* = 46 clusters) or usual care alone (*n* = 46 clusters). All 92 clusters provided breastfeeding outcome data on 9,207 mother-infant pairs (4,973 in the NOSH group, 4324 in the control group) (Table 2).

The primary outcome was the electoral ward area-level 6 to 8 weeks breastfeeding prevalence, as assessed by clinicians at the routine 6 to 8 weeks postnatal check. This was derived from the number of new mothers who were breastfeeding or not at 6 weeks in each local authority area/cluster**.** A cluster-level approach was used to analyse the primary outcome after obtaining a summary measure for each cluster. Specifically, a weighted multiple linear regression model was used in the original study.

### Analysis

The sample size characteristics of our case studies are summarised using frequencies and percentages, and all the models were fitted using complete cases. Across the case studies, the range of the missing data was from 0 to 7% which is negligible, hence no sensitivity analysis was conducted. In clinical trials, it is not uncommon to fit both unadjusted and adjusted regression models [58]. We fitted both unadjusted and adjusted models with the four analytical approaches – GLMM (with MLE and REML), GEE1, GEE2, and QIF. The unadjusted models contained only the indicator variable ${x}_{1i}$ for the randomised treatment arms as a covariate. While the adjusted models included other known prognostic covariates ${{\varvec{X}}}_{pij}^{T}$ (with the treatment arm indicator inclusive), such as baseline outcome values, age, and sex. There are several known benefits from adjusting for prognostic covariates in an adjusted analysis, such as protection against imbalance in baseline participant prognostic covariates among groups [59], increased power and precision for linear models [1, 59, 60], to obtain an estimate of the intervention effect that has a closer individual level interpretation, and to account for special features of the study design like stratification and subgroup consideration [61]. A study used simulations to show that adjusting for prognostic and non-prognostic covariates led to increased and reduced power, respectively [59].

To analyse the outcome data from the trials with few clusters we fitted a GLMM (with REML). Most small sample corrections are not compatible with MLE, hence REML was used with Satterthwaite (SAT) correction [62] applied to correct its DoF of the GLMM. Corrections on the DoF of a parameter estimate only affect the *P*-value and CI, but the point estimate of the intervention effect remains the same as that of the uncorrected version [21]. For GEE1, Fay and Graubard (FG) correction [63] was applied to correct the robust SE of the estimate of the intervention effect, which consequently affected its *P*-value and CI. All the corrections used are available in R and SAS. Although small sample corrections exist for GEE2 [16] and QIF [64], they are not readily available or easy to implement in standard statistical packages, respectively, as at the time of authoring this paper.

### Software

SAS (version 9.4) and R (version 1.4.1717) statistical software packages were used for this study. GLMM and QIF models were fitted using SAS while GEE1 and GEE2 models were fitted using R. The SAS syntax and R codes for fitting all the statistical models applied to one case study (the PoNDER trial) are provided (see, Additional file 4).

The GLMM models were fitted using the *GLIMMIX* procedure in SAS and we set the quadrature points (nodes) to 10 for the AGHQ algorithm. Higher nodes increase the complexity of the AGHQ procedure but produce more reliable results than lower nodes [26]. SAS PROC *GLIMMIX* does not produce a value for the ICC, so we calculated it using the estimates of the between cluster variation and individual variation from the *PROC GLIMMIX* GLMM output.

The QIF models were fitted using the *qif* macro in SAS. In the GEE2 models, no covariate was adjusted for the working correlation and scale parameters. The link function for the mean structure was either identity for a continuous or logit for a binary outcome, for the scale structure it was the identity, and for the correlation structure modified Fisher’s z transformation was used. GEE1 models were fitted using the *geeglm* function of R’s *geepack* package with an exchangeable correlation structure, and so was GEE2 using the *geese* function.

## Results

We assumed an exchangeable working correlation structure for all PAMs in this study, which is reasonable for a CRCT design, and it is the most assumed working correlation structure in CRCTs [31, 65]. Although the LMM was used to analyse all continuous outcomes, we labelled its results as GLMM for simplicity. In each analysis, we consider a *P *-value $<0.05$ to mean that the result is statistically significant. The results for each of the four CRCTs are presented below.

### PoNDER trial

It is worth noting the key features of the PoNDER trial [54]. The PoNDER trial had many clusters (~ 100) with an average cluster size of twenty-seven. Two outcomes were analysed, the mean EPDS score at six months (continuous) and EPDS score < or $\ge$ 12 at six months (binary), multiple covariates were adjusted for in the adjusted modelling including the baseline outcome covariate. The focus is to investigate and discuss (see, Discussion Section for more) the impact of these features on the parameter estimates from the different statistical methods.

The mean age of all the women in the control and intervention groups was the same (32 $\pm$ 5yrs, respectively), and the maximum age for all women was 46 years. The proportion of women with EPDS score $\ge$ 12 at 6 months was 16% (150/914) in the control arm and 12% (205/1745) in the intervention arm. For the continuous outcome “the mean EPDS score at six months”, was 6.4(SD = 5.0) vs. 5.5(SD = 4.9) for the control vs the intervention arms, respectively. It is worth noting that for both outcomes, smaller is better. The estimates of the unadjusted intervention effect from the analysis of the continuous primary outcome are the same across the models (mean difference = -1.00), except for QIF (-0.94). After adjustments were made for the baseline EPDS 6 weeks score, living alone, previous history of major life events, and previous history of postnatal depression, the estimates of the intervention effect became the same across the models (mean difference = -0.8, 1 d.p).

**Fig. 2:**
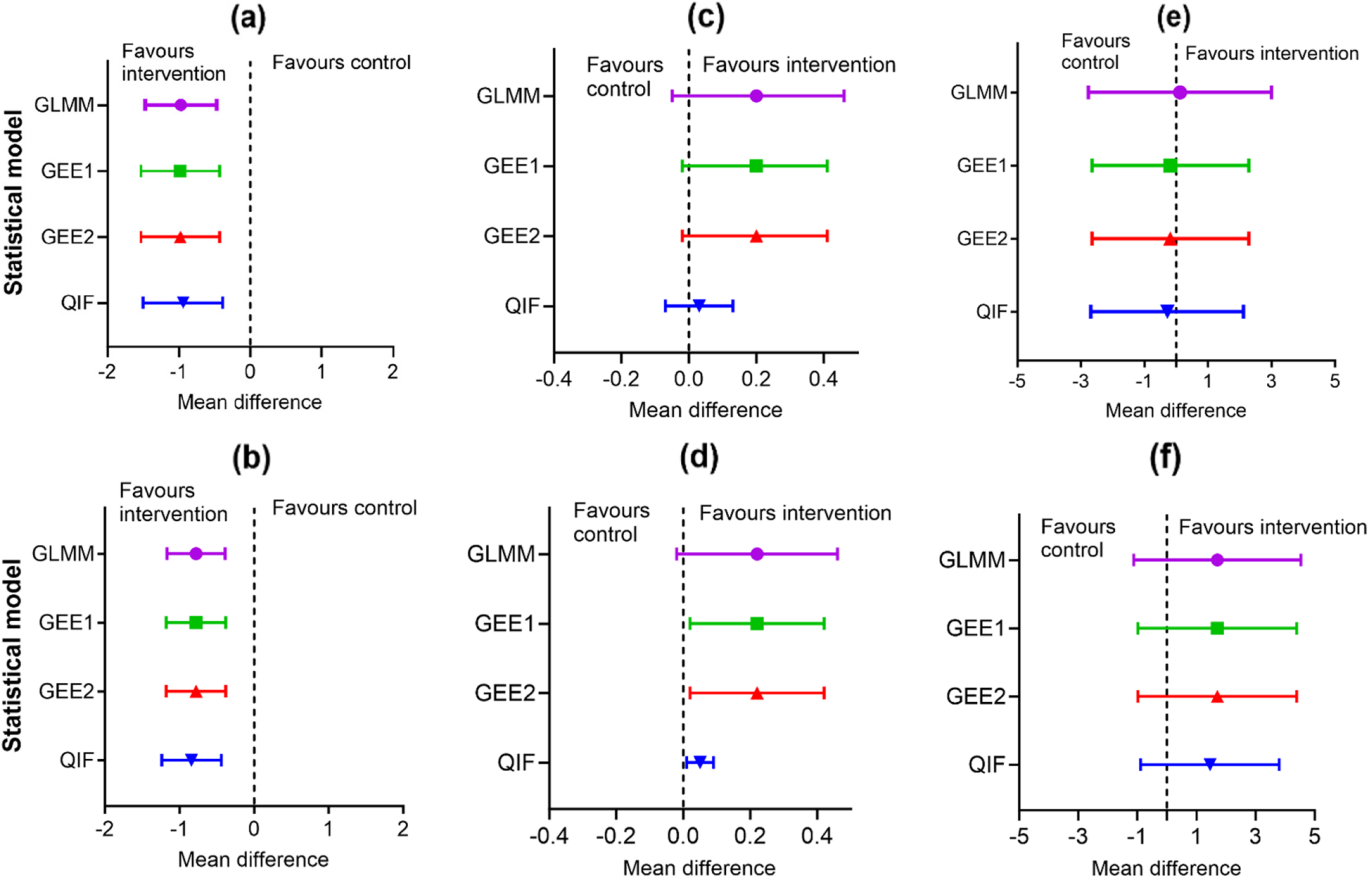
Forest plots showing the intervention effect estimate and its associated 95% CI for the four statistical models fitted using the continuous outcomes of three of the four CRCTs, where plots (**a**) & (**b**) are the unadjusted and the adjusted models fitted on the outcome data from PoNDER trial respectively, (**c**) and (**d**) is that of Informed choice and (**e**) & (**f**) is that of Bridging the Age Gap trial. The electronic version is in colour

**Fig. 3:**
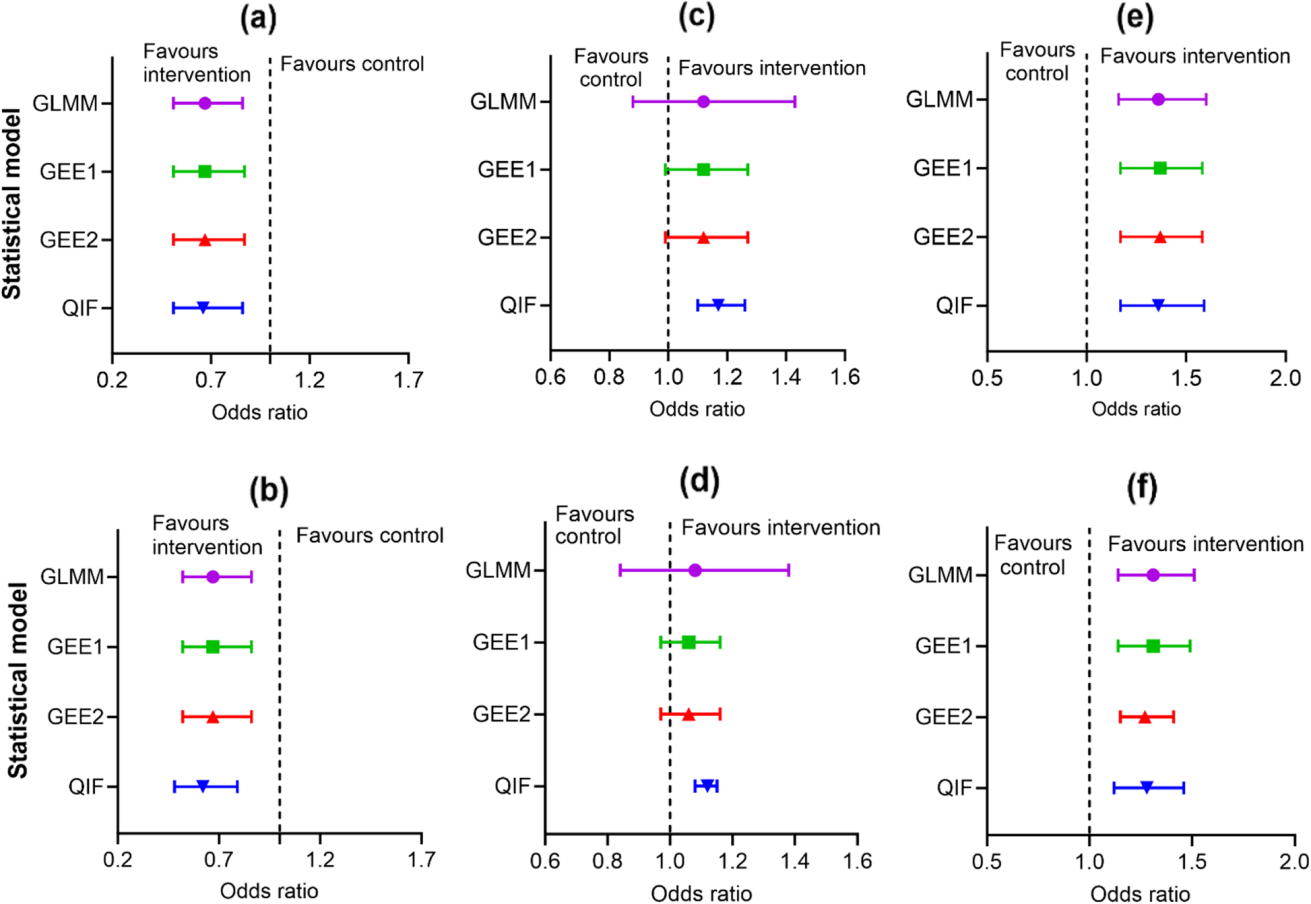
Forest plots showing the intervention effect estimate and its associated 95% CI for each of the statistical model fitted on the binary outcomes of three cluster trials datasets where plots (**a**) & (**b**) are the unadjusted and the adjusted models fitted to the outcome data from PoNDER trial respectively, (**c**) and (**d**) is that of the Informed Choice trial, and (**e**) & (**f**) is for the NOSH trial. Electronic version is in colour

The SEs of the intervention effect estimates were the same across the models, 0.3, for the unadjusted models and 0.2 for the adjusted models. The intervention effect estimates across the models were significant as evidenced by the small *P*-values (< 0.05) and the confidence intervals which excluded zero. Similar results were obtained from the binary outcome analysis, the odds ratio was approximately 0.7 across unadjusted and adjusted models, except for the adjusted QIF model (Odds ratio = 0.6). All the results were statistically significant, suggested by their small *P*-values and CIs that excluded one (Table 3). Adjusting covariates in the logistic models did not affect the magnitude of the estimates of the intervention effect from the different models, except QIF (though slightly). These results are graphically compared using forest plots and shown in Fig. 2(a, b) and Fig. 3(a, b). Looking at the plots all the point estimates for the intervention effect and the associated 95% confidence intervals (CIs) are to the left-hand side of zero favouring the intervention arm. The width of the whiskers that represent the 95% CIs is approximately the same for all the models.

**Table 3:** A summary of the results obtained from fitting the different statistical models to the PoNDER trial data (*N* = 2659)

		**Continuous outcome**^**1**^				**Binary outcome**^**2**^			
**Parameter**	**Type of modelling**	**GLMM**	**GEE1**	**GEE2**	**QIF**	**GLMM**	**GEE1**	**GEE2**	**QIF**
**Intervention effect**^**3**^	Unadjusted	-0.97	-0.98	-0.98	-0.94	0.67	0.67	0.67	0.66
	Adjusted^a^	-0.78	-0.78	-0.78	-0.84	0.67	0.67	0.67	0.62
**SE**	Unadjusted	0.25	0.28	0.28	0.28	0.13	0.14	0.14	0.14
	Adjusted^a^	0.20	0.21	0.21	0.20	0.13	0.13	0.13	0.13
***P*-value **	Unadjusted	0.0002	0.0005	0.0005	0.0009	0.0025	0.0032	0.0032	0.0019
	Adjusted^a^	0.0001	0.0001	0.0001	< 0.0001	0.0019	0.0019	0.0019	0.0001
**95% CI**	Unadjusted	-1.47 to -0.47	-1.53 to -0.43	-1.53 to -0.43	-1.50 to -0.39	0.51 to 0.86	0.51 to 0.87	0.51 to 0.87	0.51 to 0.86
	Adjusted^a^	-1.17 to -0.39	-1.18 to -0.38	-1.18 to -0.38	-1.24 to -0.44	0.52 to 0.86	0.52 to 0.86	0.52 to 0.86	0.48 to 0.79
**ICC**	Unadjusted	0.0167	0.0191	0.0382	0.0191	0.0167	0.0063	0.0126	0.0063
	Adjusted^a^	0.0077	0.0081	0.0162	0.0081	0.0000	-0.0018	-0.0036	-0.0018
**Number of subjects**	Unadjusted	2659	2659	2659	2659	2659	2659	2659	2659
	Adjusted^a^	2624	2624	2624	2624	2624	2624	2624	2624
**Number of clusters**	Unadjusted	100	100	100	100	100	100	100	100
	Adjusted^a^	100	100	100	100	100	100	100	100

^a^Model adjusted for EPDS score at 6 weeks, living alone (no or yes), previous history of major life events (no or yes), and any previous history of postnatal depression (no or yes). Note that *SE* Standard error, *CI* Confidence interval, *ICC* Intracluster correlation coefficient. *GLMM* Generalized linear mixed model, *mGLM* Marginal generalized linear model, *GEE* Generalized estimating equations, *QIF* Quadratic inference function

1. EPDS score at 6 months postnatally. The EPDS is scored on a 0 to 30 scale with higher scores indicating a greater risk of PND

2. Dichotomised EPDS score at 6 months postnatally of < 12 or $\ge$ 12

3. The intervention effect for the continuous outcome is the difference in the mean 6-month EPDS scores between the intervention and control groups; with a negative mean difference favouring lower scores (better outcomes) in the intervention group. The intervention effect for the binary outcome is the odds ratio for an EPDS score of 12 or more in the intervention group compared to the control group with an odd ratio < 1 favouring better outcomes (lower odds of PND) in the intervention group

### Informed choice trial

The Informed Choice trial had a few clusters (ten clusters) with a large average cluster size (cluster mean = 155). The analysed outcomes were “proportion of those who answered yes about making an informed choice (binary)” and “the averaged level of a woman’s knowledge about informed choice (continuous)”, and several covariates were adjusted for but none was the baseline outcome variable as this was not measured [55]. Here the interest is the impact of a small number of clusters on the estimates from the different models. In the intervention arm, 59% (477/816) of the women reported having exercised informed choice while using the maternity service compared to 57% (346/612) in the control arm. The mean knowledge of the 10 topics covered in the survey was 3.6 (SD = 1.62) for the intervention arm compared to 3.3 (SD = 1.60) for the control arm.

The results of the unadjusted and adjusted models from the analysis of the continuous and binary outcomes are presented in Table 4 and visualised in Fig. 2(c,d) and Fig. 3(c,d), respectively. For the continuous outcome, the unadjusted intervention effect estimates were the same for the three models (mean difference = 0.20, SE = 0.11), except for QIF (0.03, SE = 0.05). Similarly, the adjusted intervention effect estimates were the same 0.22 (SE = 0.1) for all the models except for QIF 0.05 (SE = 0.02). The intervention effect estimate from the QIF model is far more inconsistent with the observed data (difference in mean score = 0.3). The unadjusted intervention effects were not significant (i.e., *P* > 0.05), but the adjusted intervention effects were somewhat significant (i.e., *P* $<0.05$) except for GLMM.

**Table 4:** A summary of the results obtained from fitting the different statistical models to Informed Choice postnatal trial data (*N* = 1547)

		**Continuous outcome**^**1**^				**Binary outcome**^**2**^			
**Parameter**	**Type of modelling**	**GLMM**	**GEE1**	**GEE2**	**QIF**	**GLMM**	**GEE1**	**GEE2**	**QIF**
**Intervention effect**^**3**^	**Unadjusted**	0.20	0.20	0.20	0.03	1.12	1.12	1.12	1.17
	**Adjusted**^a^	0.22	0.22	0.22	0.05	1.08	1.06	1.06	1.12
**SE**	**Unadjusted**	0.11	0.11	0.11	0.05	0.11	0.06	0.06	0.04
	**Adjusted**^a^	0.10	0.10	0.10	0.02	0.11	0.05	0.05	0.07
***P-value***	**Unadjusted**	0.1030	0.0730	0.0731	0.5306	0.3178	0.0647	0.0647	< 0.0001
	**Adjusted**^a^	0.0676	0.0324	0.0324	0.0158	0.5206	0.2175	0.2175	< 0.0001
**95% CI**	**Unadjusted**	-0.05 to 0.46	-0.02 to 0.41	-0.02 to 0.41	-0.07 to 0.13	0.88 to 1.43	0.99 to 1.27	0.99 to 1.27	1.10 to 1.26
	**Adjusted**^a^	-0.02 to 0.46	0.02 to 0.42	0.02 to 0.42	0.01 to 0.09	0.84 to 1.38	0.97 to 1.16	0.97 to 1.16	1.08 to 1.15
**ICC**	**Unadjusted**	0.0042	0.0027	0.0055	0.0027	0.0000	-0.0029	-0.0058	-0.0029
	**Adjusted**^a^	0.0029	0.0018	0.0036	0.0018	0.0000	-0.0036	-0.0072	-0.0032
**Number of subjects**	**Unadjusted**	1534	1534	1534	1534	1485	1485	1485	1485
	**Adjusted**^a^	1474	1474	1474	1474	1439	1439	1439	1439
**Number of clusters**	**Unadjusted**	10	10	10	10	10	10	10	10
	**Adjusted**^a^	10	10	10	10	10	10	10	10

^a^Model adjusted for mother’s age, age mother left education, parity, and delivering style. Note that *SE* Standard error, *CI* Confidence interval, *ICC* Intracluster correlation coefficient, *GLMM* Generalized linear mixed model, *mGLM* Marginal generalized linear model, *GEE* Generalized estimating equations, *QIF* Quadratic inference function

1. Knowledge of informed choice leaflets score at 8 weeks postnatally. Knowledge is scored on a 0 to 10 scale with higher scores indicating greater knowledge of the leaflets

2. Proportion of women who answered “yes” to the question “Have you had enough information and discussion with midwives or doctors to make a choice together about all the things that happened during maternity care?” with the options “yes,” “partly,” “no,” “there was no choice,” and “did not apply”

3. The intervention effect for the continuous outcome is the difference in the mean 6-week knowledge scores between the intervention and control groups; with a positive mean difference favouring (better outcomes) in the intervention group. The intervention effect for the binary outcome informed choice (yes or no) is the odds ratio for yes to overall informed choice in the intervention group compared to the control group with an odds ratio > 1 favouring better outcomes (higher odds of an informed choice) in the intervention group

Similarly, for the binary outcome, the unadjusted odds ratio of women who reported exercising informed choice in the intervention arm compared to the control arm was the same for all the models (odds ratio = 1.12, SE = 0.10 to 0.11) except for QIF (1.17, SE = 0.04). The adjusted odds ratios from all the models are the same (odds ratio = 1.1, SE = 0.10 to 0.11). The unadjusted and adjusted odds ratio were not significant for all the models except that of QIF which was highly significant (*P* < 0.0001) (see, Table 4).

The results of applying small sample corrections are summarised in Table 5. When compared to the results from the uncorrected version in Table 4, the differences lie in the *P*-values and 95% CIs of the treatment effect estimates, for both the continuous and binary outcomes. The corrected *P*-values are bigger, and the CIs are wider (Table 5).

**Table 5:** A summary of the results from GLMM and GEE1 in conjunction with small sample corrections applied to the Informed Choice cRCT data (with ten clusters)

		**Continuous outcome**^**1**^				**Binary outcome**^**2**^			
**Method**	**Type of modelling**	**Intervention effect**	**SE**	***P-value***	**95% CI**	**Intervention effect**	**SE**	***P-value***	**95% CI**
**GLMM**_**Sat**_	**Unadjusted**	0.20	0.11	0.1371	(-0.09, 0.52)	1.12	0.11	0.4796	(0.29, 4.31)
	**Adjusted**^a^	0.22	0.10	0.0930	(-0.05, 0.52)	1.08	0.11	0.6234	(0.27, 4.26)
**GEE1**_**FG**_	**Unadjusted**	0.20	0.11	0.1853	(-0.13, 0.53)	1.12	0.06	0.3229	(0.79, 1.61)
	**Adjusted**^a^	0.22	0.10	0.1086	(-0.06, 0.50)	1.06	0.05	0.5495	(0.80, 1.38)

^a^Model adjusted for mother’s age, age mother left education, parity, and delivering style. Note that *SE* Standard error, *CI* Confidence interval, *GLMM* Generalized linear mixed model, *GEE* Generalized estimating equations, *QIF* Quadratic inference function, *Sat* Satterthwaite, *FG* Fay & Graubard

1. Knowledge of informed choice leaflets score at 8 weeks postnatally. Knowledge is scored on a 0 to 10 scale with higher scores indicating a greater knowledge of the leaflets

2. Proportion of women who answered “yes” to the question “Have you had enough information and discussion with midwives or doctors to make a choice together about all the things that happened during maternity care?” with the options “yes,” “partly,” “no,” “there was no choice,” and “did not apply.”

### Bridging the age gap trial

The key features of Bridging the Age Gap trial are, a moderate number of clusters (forty-three clusters) with an average size of eighteen, the continuous outcome measured was global health status/quality of life at six months (measured at baseline and follow-up periods) [56]. The focus is on how the moderate number of clusters (and moderate average cluster size) and baseline outcome values affected the estimates from the four different statistical methods.

Table 6 presents the results from the analysis of the continuous outcome data, which is graphically shown in Fig. 2(e,f). The mean global health status/quality of life (QoL) score at the 6-month follow-up was 68.9 (SD 19.6) for the control arm against 69.0 (SD 19.5) for the intervention arm. The unadjusted models produced different estimates of the intervention effect ranging from a mean difference of -0.28 to 0.12 but became stable and changed direction after the baseline QoL variable (*ql scale*) was adjusted for; the mean difference became 1.71 for all the models except QIF (mean difference = 1.46). However, the SEs of the treatment effect estimates from GEE1 and GEE2 increased while that of the GLMM and QIF reduced after the baseline outcome covariate adjustment. The SEs are approximately the same for the adjusted models (1.40) except for QIF (1.20). All the SE estimates from QIF were lesser compared to the other three models, lesser SE is indicative of better precision provided that the method is not biased towards the null [66]. Hence, the results from QIF should be interpreted with caution, because QIF produced different estimates of the intervention effect compared to the other three models which could be indicative of biasedness. Nonetheless, none of the intervention effect estimates was significant (i.e., *P* > 0.05).

**Table 6:** A summary of the results from the models fitted to the continuous outcome data from Bridging the Age Gap trial ^1^ (*N* = 748)

	**Unadjusted model**				**Adjusted model**^a^			
**Parameters**	**GLMM**	**GEE1**	**GEE2**	**QIF**	**GLMM**	**GEE1**	**GEE2**	**QIF**
**Intervention effect**^**2**^								
	0.12	-0.19	-0.19	-0.28	1.71	1.71	1.71	1.46
**SE**								
	1.43	1.26	1.26	1.23	1.40	1.37	1.37	1.20
***P-value***								
	0.9343	0.8818	0.8810	0.8175	0.2294	0.2127	0.2127	0.2230
**95% CI**								
	-2.77 to 3.00	-2.65 to 2.28	-2.65 to 2.28	-2.69 to 2.12	-1.12 to 4.53	-0.98 to 4.39	-0.98 to 4.39	-0.89 to 3.80
**ICC**								
	0.0000	-0.0068	-0.0135	-0.0068	0.0042	0.0028	0.0056	0.0028
**Number of subjects**								
	748	748	748	748	712	712	712	712
**Number of clusters**								
	43	43	43	43	43	43	43	43

^a^Model adjusted for global QoL baseline outcome values. Note that SE Standard error; *CI* Confidence interval, *ICC* Intracluster correlation coefficient, *GLMM* Generalized linear mixed model, *mGLM* Marginal generalized linear model, *GEE* Generalized estimating equations, *QIF* Quadratic inference function

1. Global QoL score on the EORTC-C30 at 6 months post-baseline. The EORTC-C30 Global scale is scored on a 0 (poor) to 100 (good health) scale

2. The intervention effect for the continuous outcome is the difference in the mean 6-month Global QoL scores between the intervention groups; with a positive mean difference favouring higher scores (better outcomes) in the intervention group

### The NOSH trial

In this study, only binary outcome was measured (i.e., the prevalence of breastfeeding in the electoral ward assessed during the routine 6–8 week postnatal check), and the number of clusters randomised was large (Ninety-two clusters) [57]. The adjusted models included cluster-level baseline outcomes and local government areas as covariates. The unique feature of this trial is that only cluster-level covariates were adjusted for.

The results from the unadjusted and adjusted models are presented in Table 7 and are graphically presented in Fig. 3(e, f). Overall, 36% (1869/4973) of mothers in the 46 clusters of the NOSH group were breastfeeding at 6 weeks compared to 30% (1299/4324) in the 46 clusters of the control group. The odds ratios that the mothers were breastfeeding at the end of the trial were approximately the same for all the unadjusted (1.40) and adjusted (1.30) models and were statistically significant. However, it is only in this trial that the intervention effects of GEE1 and GEE2 were different, in the other trials presented previously they were the same. The SEs of the unadjusted intervention effect estimate (SEs, 0.08) and the adjusted version (SEs, 0.07) were the same for all the models, except for the adjusted GEE2 (0.05).

**Table 7:** A summary of the results obtained from fitting the different statistical models to the binary outcome data from NOSH CRCT(*N* = 9207)

	**Unadjusted model**				**Adjusted model**^a^			
**Parameters**	**GLMM**	**GEE1**	**GEE2**	**QIF**	**GLMM**	**GEE1**	**GEE2**	**QIF**
**Intervention effect**^**2**^								
	1.37	1.36	1.36	1.36	1.31	1.31	1.27	1.28
**SE**								
	0.08	0.08	0.08	0.08	0.07	0.07	0.05	0.07
***P***-**value **								
	0.0002	< 0.0001	< 0.0001	0.0009	0.0002	< 0.0001	< 0.0001	0.0002
**95% CI**								
	1.16 to 1.60	1.17 to 1.58	1.17 to 1.58	1.17 to 1.59	1.14 to 1.51	1.14 to 1.49	1.15 to 1.41	1.12 to 1.46
**ICC**								
	0.0262	0.0192	0.0383	0.0192	0.0162	0.0098	0.0042	0.0098
**Number of subjects**								
	9207	9207	9207	9207	9207	9207	9207	9207
**Number of clusters**								
	92	92	92	92	92	92	92	92

^a^The statistical models were adjusted for the cluster-level baseline breastfeeding rate and local government area. Note that *SE* Standard error, *CI* Confidence interval, *ICC* Intracluster correlation coefficient, *GLMM* Generalized linear mixed model, *mGLM* Marginal generalized linear model, *GEE* Generalized estimating equations, *QIF* Quadratic inference function

1. The binary outcome was if the mother was breastfeeding her baby at 6 weeks postnatally (response value = 1) or not (response value = 0)

2. The intervention effect for the binary outcome is the odds for breastfeeding at 6 weeks postnatally in the NOSH intervention group compared to the odds of breastfeeding in the control group with an odds ratio > 1 favouring better outcomes (higher odds of breastfeeding) in the intervention group

## Discussion

In this paper, four different approaches for analysing CRCTs with clustering in the treatment arms have been described. The four approaches GLMM, GEE1, GEE2, and QIF have been applied to four case studies with different features to demonstrate their implementation and evaluate their use in practice. To the best of our knowledge, this is the first study to comparatively evaluate these four methods in the context of CRCTs.

The initial plan was to fit all the models using free and open software such as R, but we observed that the *qif* command in the R’s *qif package (CRAN—Package qif (r-project.org))* could not fit the QIF model to data with clusters size of one. The PoNDER and Bridging Age Gap trials have clusters of size one, the error message suggests that it is a problem of the incompatibility of the matrices in the matrix multiplication procedure. So, we switched to using SAS which was able to overcome the problem. We communicated our observation to one of the developers of the two QIF’s functions of both software packages (i.e., R and SAS), Peter X.K. Song, through email correspondence and Song promised to investigate it. Also, the *lmer* command for fitting linear mixed effects model to continuous outcomes in the *lme4* package in R does not have AGHQ as an option but *glmer* for generalized linear mixed modelling does. The SAS procedure, *GLIMMIX,* has AGHQ as an option for mixed effects models for both continuous and binary outcomes.

There are previous reviews that are similar to our current methodological review, but some differences still exist. A good example is the review by Murray et al., [67] where they discussed recent methodological advances in the design and analysis of group randomised trials [67]. They looked at a five years span starting from 1999 to 2004, and they identified and discussed advances in analytical methods such as the mixed effects models with parameters estimated by MLE/REML, GEE1, Bayesian mixed effects models, survival models based on MLE and Cox methods (with robust SE), and randomisation tests. Their paper was updated in 2017 by Turner et al., of which additional methods such as augmented GEE1 (AU-GEE1), QIF, TMLE, and permutation tests were identified [68].

Our current review is more consistent with the findings of Turner et al., [68] than that of Murray. Our review was a scoping methodological review making it more comprehensive, we also employed systematic searching techniques which resulted in more methods for analysing outcome data from CRCTs being identified (27 unique methods), such as quantile GEE1 [69], generalized least squares [70], AUGEE1—inverse probability weighted (AUGEE-IPW) [71], weighted jack-knife [70]. Under methods used to analyse time to event outcome, we found a quantile estimator [72], hierarchical likelihood [73], hierarchical likelihood Laplace [73], and two-stage estimator [74] (Table S1, see Additional file 3).

Another review focused on methods used in the analysis of outcome data from stepped wedge CRCT design [75]. Similarly, Arnup et al. [76] review was focused on crossover CRCT design and was a practice review [76], whereas, own current review was a methodological review encompassing all the different types of CRCT designs with a focus on all the available and appropriate methods. A recent methodological review by Caille et al., [77] considered only methods for analysing time-to-event outcome data in CRCTs. Hence the authors identified more survival methods than our current review, such as the log-rank test, Kaplan–Meier plots, Gray’s model, competing risk model, and Fine & Gray’s cumulative incidence curve model adjusted for clustering [77]. The case studies considered have small estimates for the ICC which are consistent with those reported in primary care [78] and community-based trials [29]. The observed ICCs were less than 0.05 and three out of the four studies had an ICC less than 0.02. This indicates that there was a low clustering of outcomes as expected from primary care and community-based CRCT [29, 78]. Three studies had negative estimates for the ICC, from GEE1, GEE2, and QIF methods (i.e., from all PAMs).

Upon reading the documentation of the functions for fitting the population average models, *geeglm (for GEE1), geese (for GEE2)* functions in *R,* and the *qif* macro in SAS we could not ascertain which of the estimators (i.e., Eq. (6) or [7]) that is being used in computing their ICC estimates. However, it is more likely that the population average models are using Eq. (7) or a method similar to [7], which could be the reason why negative ICC estimates were obtained. From a sample survey perspective, sampling error due to finite sample cluster size compared to the population cluster size which is assumed to be infinite could be the cause of the negative ICC estimates [79]. Another reason is when there are large discrepancies in the allotment of trial resources within the clusters, this would cause large variations in the observed outcomes [32], in other words, there is competition among the experimental units for the limited available resources resulting in the large variations observed within clusters.

Our results showed that estimates for the intervention effect, SE, P-value, and 95% CI were the same for GEE1 and GEE2 models in almost all cases, they only differ in their estimates for the ICC. This means that both methods fit the same models regardless of whether the correlation parameter is estimated or considered as a nuisance within the methods formulations, however, in GEE2 models the ICC parameter is explicitly modelled which could be recourse to producing a more consistent ICC estimate (i.e., adequately accounting for clustering) compared to GEE1 [10, 13], especially if the correlation is substantial.

If the observed ICC is anticipated to be large or varies by cluster sizes, it is recommended that models that allow for heterogenous correlation structure should be considered, such as GEE2, because it is likely to improve inference [10]. This happens to be the major merit of Yan & Fines’ 3EE GEE2 model [13] over GEE1. Hence, it would be worth investigating to know which of the two methods is adequately modelling the correlation within clusters, since if the correlation is large and misspecified it could cause some loss in efficiency of the intervention effect estimate (i.e., having treatment effect estimates within bigger SEs). This can be achieved through simulation studies, where the true ICC value is known. Accurate estimates of the ICC are needed for planning future cluster trials [61, 80]. Our four case studies exhibited some common features of CRCT design that are unique to primary care and community-based CRCTs. The impact of these key features on the estimates from the four statistical models is evident in the results obtained.

For example, the PoNDER trial was conducted in a primary care setting and hence had a large sample size (both in the number of clusters and cluster sizes, 100 clusters with an average cluster size of 26). Hence, the unadjusted and adjusted intervention effect estimates from the different methods were the same for the continuous and binary outcomes analyses, that of QIF were slightly different. The odds ratios obtained possibly showed the noncollapsible feature of the logistic regression model (with a logit link) – where including a baseline covariate changes the size of the intervention effect estimate, if the covariate is a strong predictor of the outcome, even if it is not related to the treatment conditions [81]. Since in this particular case the estimated intervention effect did not change upon inclusion of the baseline covariates in the adjusted analysis, except for QIF, possibly indicating that the covariates are not strong predictors of the outcome.

On the aspect of hypothesis testing, the conclusions reached were the same regardless of the statistical models used and it is consistent with findings of the original analysis by Morrell et al., [54]; a significant benefit of training health visitors to adequately manage women with postnatal depressive symptoms (i.e., favouring the intervention arm). The ICC estimates were small as expected [29, 78], and that of the population average logistic models were negative (i.e., GEE1, GEE2, and QIF). These results are consistent with the findings of Adam et al. [78], they reanalyse thirty-one CRCTs conducted within primary care settings and provided ICC estimates for several common variables. Their median unadjusted ICC was 0.01 while the adjusted was 0.005. Similarly, our results are consistent with previous simulation studies, the studies found that both cluster-specific models (typified by GLMM) and population average models (typified by GEE1) produced similar results for CRCTs that have many clusters and small ICC with binary [18] or continuous outcomes analysed [21]. Hence, for large trials with low correlation within clusters, any of the four modelling approaches (GLMM, GEE1, GEE2, and QIF) could be used. Therefore, the choice of which model to use would be based on other factors like the aim of the research.

Informed Choice trial had a few clusters (10 clusters) with a large average cluster size (median cluster size = 145). In the original study, a cross-sectional repeated measurement approach was used, so the estimate for the intervention effect was the interaction effect term between the treatment group (*group*) and time of measurement (*time*). However, for demonstration, we used only the “after intervention” postnatal sample. Both cluster and individual-level covariates were included in the adjusted models. Three of the methods produced the same estimates which differed from that of QIF, for both continuous and binary outcomes. The most obvious difference occurred in the *P*-values, CIs, and SEs (continuous outcome analysis only). For the continuous outcome, the adjusted *P*-value of GEE1 (including GEE2, and QIF) was significant whereas that of the GLMM was not (Table 4). This could indicate that the few clusters had more impact on the population average models compared to the cluster-specific model (typified by GLMM).

For binary outcome, the unadjusted and adjusted *P*-values of QIF were significant but that of the other three methods were not. This could be indicative of a possible inflated test size, and bias in the estimated intervention effect. This result is consistent with the findings of previous studies [15–17]. The QIF’s 95% CI of the intervention effect estimates were narrower compared to the other methods. Westgate and Braun [15] found that the impact of the interplay between the small number of clusters, covariates, and cluster size imbalance was more severe on QIF than GEE1. A correction was proposed to improve the empirically estimated covariance matrix that causes the QIF to be poorly behaved [17]. Also, GLMM was found to perform better than GEE1 in maintaining the nominal Type I error and power in trials with few clusters ($\le 20)$ for both continuous [21] and binary outcomes [23]. The results from this present study are consistent with these previous findings; however, it is more likely that the differing results from the QIF are due to the impact of the small number of clusters (which is a recipe for large cluster variations). Given these findings, it is likely that the QIF is severely affected by few to moderate numbers of clusters, followed by GEE1 then GLMM. Although, no simulation study has been carried out to compare these three methods in this regard, to reach a definite conclusion.

Informed Choice trial had a small number of clusters – ten clusters. Studies with small numbers of clusters have a higher risk of imbalance in covariates and outcomes across treatment arms/clusters [1, 15, 21]. Hence, for a study with a continuous outcome and clusters $\le$ 20, small sample corrections are required to maintain the nominal 5% Type I error and a reasonable power [21]. Similarly, if the study measured a binary outcome and the number of clusters randomised is $\le$ 30, a small sample correction should be applied to the DoF of GLMM, which is the number of clusters minus cluster-level parameters estimated [23]. We only applied small sample corrections in conjunction with GLMM and GEE1. Although there are recommended corrections for GEE2 [64] and QIF [16], however, they are not readily available or easy to implement in standard statistical packages, respectively. The employed small sample corrections resulted in bigger *P*-values and wider CIs of the intervention effect estimates. Our small sample correction findings are consistent with those of other studies [21, 23, 24].

Bridging the Age Gap trial had a moderate sample size (43 clusters with an average cluster size of 18 individual subjects), and small ICC estimates. Negative ICC estimates were associated with negative treatment effect estimates from the three PAMs. Theoretically, the ICC is bounded between 0 and 1. But in practice, negative ICCs can be realised from real-world data with finite samples. The GLMM model truncates the ICC to zero instead of producing a negative ICC, effectively fitting a generalized linear model (GLM) [82], but that is not the same for the other three population average models – GEE1, GEE2, and QIF [79]. Our results confirmed this, only the PAMs produced negative ICC estimates, this occurred in trials with a small to moderate number of clusters (Table 4 and Table 6). Regardless of the size of the ICC, it is ideal to use an analytical method that accounts for clustering in a CRCT. Across the four statistical models, the unadjusted intervention effect estimates were unstable ranging from -0.28 to 0.12 but became stable after the baseline outcome covariate was adjusted for (mean difference = 1.78), except for QIF (mean difference = 1.46) which also had the smallest SE estimates. This elucidates the importance of accounting for relevant prognostic factors in clinical trials, especially the baseline outcome covariate [1]. However, for linear models, covariate adjustment does not change the intervention effect estimate, although it does increase its precision (i.e., reduce the SE of the intervention effect estimate) [1]. In the case of a nonlinear model, covariates adjustment does affect the estimate of the intervention effect and also leads to reduced precision [60]. In general, for a balanced trial with a continuous outcome, the unadjusted and adjusted analyses would produce equivalent estimates, but the adjusted analysis will be more precise, especially when the covariates are strongly correlated with the outcome [1]. Hence, in most cases, for both linear and nonlinear models, adjusted analysis is mostly encouraged, however, the two are often reported [1, 60].

This was similar for the SEs and the 95% CIs of the treatment effect estimate. QIF appeared to be slightly more precise than the other methods (i.e., had smaller SEs). However, this result should be interpreted with caution since the estimate of its intervention effect could be biased – methods that are biased toward the null hypothesis often tend to have smaller SEs [66]. Studies by Westgate confirmed this possibility of QIF being negatively biased for trials with small to moderate clusters [16]. Similarly, studies have found that the GLMM with parameters estimated by REML performs better than GEE1 in maintaining the nominal Type I error rate and power, for continuous [21] and binary outcomes [23] when the number of clusters is moderate or small. Nonetheless, all four statistical models resulted in the same inference and are consistent with that of the original analysis which was “no significant difference in the Global QoL between the control and the intervention arms” [56].

Lastly, for the NOSH trial with only binary outcome measured, and a large sample size (92 clusters with an average cluster size of 100 individual subjects). The parameter estimates from the four statistical approaches are the same in almost all cases, hence, their performance was equivalent. A unique finding here is that it is only in this case study that GEE2 produced a different adjusted intervention effect estimate compared to GEE1 (1.27 vs. 1.31) with SEs of 0.05 vs. 0.07, consequently, their 95% CIs were different. The key feature of the NOSH trial which is different from other case studies is that in NOSH, only cluster-level covariates were adjusted for, maybe this feature had a differing impact on the GEE1 and GEE2. Further studies are needed to confirm this.

Our results revealed some insight into the possible simulation studies that should be conducted to investigate the operating characteristics of these four analytical approaches. Simulation studies involve generating pseudo-random numbers from computer-designed experiments that mimic different settings of CRCT design [66]. For example, two of the trials had small and moderate numbers of clusters. This feature affected QIF differently – QIF had smaller estimates for the intervention effect and its SE. A simulation study where the true parameters are known and varied to cover a reasonable parameter range should be conducted. The parameters that could be varied include the number of clusters, levels of ICC, effect sizes (i.e., the true intervention effect), cluster sizes, types of outcomes, and distribution of the cluster random. This will help create different scenarios that are needed to investigate the independent and combined impact of the varied parameters on the performance of the methods. Another possible simulation study that is similar to the one stated above, but with a focus on the impact of small numbers of clusters ($\le$ 30 clusters), and the methods would include both the uncorrected and corrected versions (corrected of the effect of small sample) of the four methods. This study will determine how well the corrected versions of the methods perform both absolutely and relatively.

### Limitations

This study employed a formal systematic search of relevant literature to capture most of the related work conducted. However, this was not an exhaustive review of all work in this area.

We have used four case studies that have arisen from our work as applied medical statisticians in clinical trial research. The results and inferences made apply to data from CRCTs with similar properties to our case studies. For example, our investigation focused on binary and continuous endpoints, studies with observed ICCs similar to trials conducted within primary care and community-based settings, used complete cases, and some having few clusters. However, this data limitation (i.e., missing data) might not result in adverse consequences since the proportions that were missing were small. Although, the other data limitations (i.e., a small number of clusters) might be.

While a small number of clusters, and incomplete data are issues in many real-world data sets, to increase the generalisability of our results to trials with different characteristics to our case studies, we hope to conduct a simulation study soon. The study will explore how our findings might change when the following parameters: cluster sizes, ICC, and number of clusters are varied.

## Conclusion

In summary, we analysed outcome data from four CRCTs to demonstrate the applications of four statistical methods that are appropriate for analysing CRCTs. The characteristics of the four case studies covered some common settings in CRCTs; however, the generalizability of our findings should be limited to studies with similar characteristics as our case studies. In most cases, the modelling approaches produced similar results which are consistent with the original analyses. This is not uncommon, because our case studies typified primary care and community based with low clustering and common sample sizes (i.e., small, moderate, and large).

In some cases, QIF produced differing estimates compared to the other three approaches. These differences are noticeable for studies with a small to moderate number of clusters (i.e., $\le$ 43). Although the four statistical methods were compared to each other, we cannot determine a superior method using only this example data analysis. Nonetheless, we recommend that for trials with a small to moderate number of clusters, caution should be exercised when QIF is used without small sample correction. It is necessary to conduct further research based on simulation studies to comprehensively evaluate the performances of the analytical approaches.

### Supplementary Information

**Additional file 1.** Search strategy.

**Additional file 2:** **Figure S1.** Trend of published papers on statistical methods for analysing outcome data from cRCTs, from January 2003 to December 2020.

**Additional file 3:** **Table S1.** The frequency of study of each statistical method for analysing outcome data from cRCTs (*N* = 112).

**Additional file 4.** SAS syntax and R code for fitting the models on PoNDER trial data set only SAS syntax.

## Data Availability

Data are available upon reasonable request from BCO at bcofforha1@sheffield.ac.uk.

## Abbreviations

IRCT: Individual randomised controlled trial
CRCT: Cluster randomised controlled trial
TMLE: Targeted maximum likelihood estimator
ALR: Alternating logistic regression
GEE: Generalized estimating equations
QIF: Quadratic inference function
MLE: Maximum likelihood estimator
LMM: Linear mixed model
GLMM: Generalized linear mixed model
mGLM: Marginal generalized linear model
GLM: Generalized linear model
REML: Restricted maximum likelihood
CLA: Cluster level analysis
ILA: Individual level analysis
AGHQ: Adaptive Gauss-Hermit quadrature
ICC: Intracluster correlation coefficient
MGF: Moment generating function
3EE: Three estimating equations
GMM: Generalized method of moments
