# Supplementary material for: Analysing cluster randomised controlled trials using GLMM, GEE1, GEE2, and QIF: results from four case studies
Source: BMC Med Res Methodol. 2023 Dec 13;23:293. doi: 10.1186/s12874-023-02107-z (PMC10717070; doi:10.1186/s12874-023-02107-z)
Supplement: Supplementary file 1 — Additional file 1. Search strategy. [file 12874_2023_2107_MOESM1_ESM.docx]

**APPENDIX A**

**Search strategy**

***Search terms***Medline/PsycInfo/EMbase via Ovid:
1. (statistic* model* or statistic* method*).mp.
2. (group randomi* or community randomi*).ti.
3. cluster.tw.
4. 2 or 3
5. randomi* controlled trial.ti.
6. randomi*.mp.
7. placebo.mp.
8. 5 or 6 or 7
9. 1 and 4 and 8

Scopus and Cinahl (via Ebsco):
1. “statistic* model*” or “statistic* method*”
2. “group randomi*” or “community randomi*”
3. cluster
4. 2 or 3
5. randomi* controlled trial
6. randomi*
7. placebo
8. 5 or 6 or 7
9. 1 and 4 and 8
Limiters:
1. Language: English only
2. Time frame: from 1st January 2003 to 19th December 2020.
