# Supplementary material for: Analysing cluster randomised controlled trials using GLMM, GEE1, GEE2, and QIF: results from four case studies
Source: BMC Med Res Methodol. 2023 Dec 13;23:293. doi: 10.1186/s12874-023-02107-z (PMC10717070; doi:10.1186/s12874-023-02107-z)

# **APPENDIX B**

**Figure S1** Trend of published papers on statistical methods for analysing outcome data from cRCTs,

from January 2003 to December 2020.


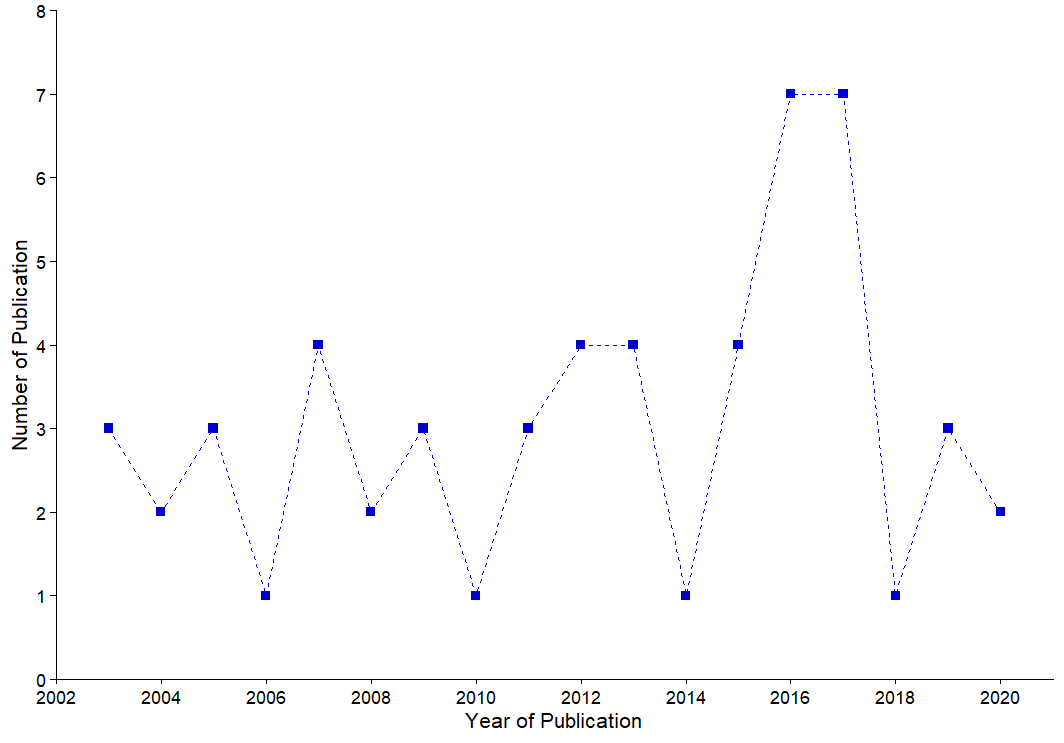

Supplement: Supplementary file 2 — Additional file 2: Figure S1. Trend of published papers on statistical methods for analysing outcome data from cRCTs, from January 2003 to December 2020. [file 12874_2023_2107_MOESM2_ESM.docx]
