# Supplementary material for: Analysing cluster randomised controlled trials using GLMM, GEE1, GEE2, and QIF: results from four case studies
Source: BMC Med Res Methodol. 2023 Dec 13;23:293. doi: 10.1186/s12874-023-02107-z (PMC10717070; doi:10.1186/s12874-023-02107-z)
Supplement: Supplementary file 3 — Additional file 3: Table S1. The frequency of study of each statistical method for analysing outcome data from cRCTs (N = 112). [file 12874_2023_2107_MOESM3_ESM.docx]

**APPENDIX C**

**Table S1** The frequency of study of each statistical method for analysing outcome data from cRCTs (N = 112)

|  | | |  |  |  |  |
| --- | --- | --- | --- | --- | --- | --- |
| **Statistical method** | **n** | **Reference** | | | |  |
| **Cluster-level analysis** | | | | | | |
| Generalized least squares (GLS) | 1 | (1) | | | |  |
| Weighted Jack-knife | 1 | (1) | | | |  |
| t-test | 7 | (2–8) | | | |  |
| Wilcoxon rank sum test | 2 | (2,9) | | | |  |
| Kruskal Wallis | 1 | (10) | | | |  |
| Permutation test | 3 | (2,11,12) | | | |  |
| Ordinary least squares (OLS) | 4 | (6,13–15) | | | |  |
| Weighted least squares | 2 | (16,17) | | | |  |
| Restricted MLE (REML) | 1 | (17) | | | |  |
| **Individual-level analysis** | |  | | | |  |
| Quadratic inference function (QIF) | 5 | (18–22) | | | |  |
| First-order generalized estimating equations (GEE1) | 23 | (2–8,10,15,18–20,22–32) | | | |  |
| Corrected orthogonalized residual | 1 | (33) | | | |  |
| Quantile-GEE1 | 1 | (34) | | | |  |
| AUGEE-IPW^†^ | 1 | (35) | | | |  |
| Maximum likelihood estimation (MLE) | 18 | (8,10,15,23–26,30,36–45) | | | |  |
| REML | 11 | (6,13,15,16,31,32,36,41,45–47) | | | |  |
| Iteratively reweighted least squares (IWLS) | 1 | (47) | | | |  |
| Multivariate penalized likelihood (MPL) | 1 | (44) | | | |  |
| Hierarchical likelihood (HL) | 2 | (38,48) | | | |  |
| Hierarchical likelihood-Laplace (HLA) | 1 | (38) | | | |  |
| Penalized quasi-likelihood (PQL) | 3 | (23,26,40) | | | |  |
| Pseudo-likelihood (PL) | 1 | (5) | | | |  |
| Pseudo-likelihood – risk set (PLRS) | 1 | (49) | | | |  |
| Adjusted Chi-square test | 3 | (2,7,10) | | | |  |
| Two-stage estimator | 1 | (50) | | | |  |
| Quantile estimator | 1 | (51) | | | |  |
| Bayesian methods (with MCMC) | 13 | (5,15,37,40,43,52–59) | | | |  |
| Ordinary least squares (OLS) | 1 | (32) | | | |  |
| Targeted maximum likelihood estimation (TMLE) | 1 | (60) | | | |  |

N = The total number of times the methods were studied, n = The number of times each method was studied. ^†^AUGEE-IPW: Augmented generalized estimating equations – inverse probability weighted.

**REFERENCES**

1. Du R, Lee JH. A weighted Jackknife method for clustered data. Commun Stat - Theory Methods. 2019;48(8):1963–80.

2. Austin PC. A comparison of the statistical power of different methods for the analysis of cluster randomization trials with binary outcomes. Stat Med. 2007;26(Jan):3550-3565-3550–65.

3. Austin PC. A comparison of the statistical power of different methods for the analysis of repeated cross-sectional cluster randomization trials with binary outcomes. Int J Biostat [Internet]. 2010;6(1). Available from: https://www.scopus.com/inward/record.uri?eid=2-s2.0-77950524668&doi=10.2202%2F1557-4679.1179&partnerID=40&md5=ca4acf77300c58dbe435f7e86fc641f1

4. Ukoumunne OC, Forbes AB, Carlin JB, Gulliford MC. Comparison of the risk difference, risk ratio and odds ratio scales for quantifying the unadjusted intervention effect in cluster randomized trials. Stat Med. 2008 Nov;27(25):5143–55.

5. Pacheco GD, Hattendorf J, Colford Jr. JM, Mäusezahl D, Smith T. Performance of analytical methods for overdispersed counts in cluster randomized trials: Sample size, degree of clustering and imbalance. Stat Med. 2009;28(24):2989–3011.

6. Walters SJ, Morrell CJ, Slade P. Analysing data from a cluster randomized trial (cRCT) in primary care: A case study. J Appl Stat. 2011;38(10):2253–69.

7. Peek N, Goud R, De Keizer N. Handling intra-cluster correlation when analyzing the effects of decision support on health care process measures. In Dept. of Medical Informatics, University of Amsterdam, PO Box 22700, 1100 DD Amsterdam, Netherlands; 2013. p. 22–7. Available from: https://www.scopus.com/inward/record.uri?eid=2-s2.0-84886769645&doi=10.3233%2F978-1-61499-240-0-22&partnerID=40&md5=e7bf1d8a748ede3ab90d55aa59e00689

8. Hossain A, Bartlett JW. Missing binary outcomes under covariate-dependent missingness in. Stat Methods Med Res. 2017;36(19):3092–109.

9. Leyrat C, Morgan KE, Leurent B, Kahan BC. Cluster randomized trials with a small number of clusters: Which analyses should be used? Int J Epidemiol. 2018;47(1):321–31.

10. Kim HY, Preisser JS, Rozier RG, Valiyaparambil JV. Multilevel analysis of group-randomized trials with binary outcomes. Community Dent Oral Epidemiol. 2006;34(4):241–51.

11. Murray DM, Hannan PJ, Pals SP, McCowen RG, Baker WL, Blitstein JL. A comparison of permutation and mixed-model regression methods for the analysis of simulated data in the context of a group-randomized trial. Stat Med. 2006;25(3):375–88.

12. Wang R, De Gruttola V. The use of permutation tests for the analysis of parallel and stepped-wedge cluster-randomized trials. Stat Med. 2017;36(18):2831–43.

13. Du R, Lee JH. A weighted Jackknife method for clustered data. Commun Stat - Theory Methods. 2019;48(8):1963–80.

14. Ukoumunne OC, Carlin JB, Gulliford MC. A simulation study of odds ratio estimation for binary outcomes from cluster randomized trials. Stat Med. 2007;26(18):3415–28.

15. Mcneish D, Stapleton LM, Mcneish D, Stapleton LM. Modeling Clustered Data with Very Few Clusters Modeling Clustered Data with Very Few Clusters. Multivar Behav Res. 2016;51(4):495–518.

16. Johnson JL, Kreidler SM, Catellier DJ, Murray DM, Muller KE, Glueck DH, et al. Recommendations for choosing an analysis method that controls Type I error for unbalanced cluster sample designs with Gaussian outcomes. Stat Med. 2015;34(27):3531–45.

17. Ukoumunne OC, Carlin JB, Gulliford MC. A simulation study of odds ratio estimation for binary outcomes from cluster randomized trials. Stat Med. 2007;26(18):3415–28.

18. Westgate PM. A bias-corrected covariance estimate for improved inference with quadratic inference functions. Stat Med. 2012;31(29):4003–22.

19. Westgate PM, Braun TM. The effect of cluster size imbalance and covariates on the estimation performance of quadratic inference functions. Stat Med. 2012;31(20):2209–22.

20. Westgate PM, Braun TM. An improved quadratic inference function for parameter estimation in the analysis of correlated data. Stat Med. 2013;32(19):3260–73.

21. Yang W, Liao S. A study of quadratic inference functions with alternative weighting matrices. Commun Stat---Simul Comput-275pt. 2017;46(2):994–1007.

22. Yu H, Li F, Turner EL. An evaluation of quadratic inference functions for estimating intervention effects in cluster randomized trials. Contemp Clin Trials Commun. 2020;19:100605–100605.

23. Heo M, Leon AC. Comparison of statistical methods for analysis of clustered binary observations. Stat Med. 2005;24(6):911–23.

24. Ma J, Thabane L, Kaczorowski J, Chambers L, Dolovich L, Karwalajtys T, et al. Comparison of Bayesian and classical methods in the analysis of cluster randomized controlled trials with a binary outcome: The Community Hypertension Assessment Trial (CHAT). BMC Med Res Methodol. 2009 Dec;9(1):37.

25. Ma J, Raina P, Beyene J, Thabane L. Comparison of population-averaged and cluster-specific models for the analysis of cluster randomized trials with missing binary outcomes: a simulation study. BMC Med Res Methodol. 2013;13:9.

26. Yelland LN, Salter AB, Ryan P, Makrides M. Analysis of binary outcomes from randomised trials including multiple births: When should clustering be taken into account? Paediatr Perinat Epidemiol. 2011;25(3):283–97.

27. Yelland LN, Sullivan TR, Pavlou M, Seaman SR. Analysis of Randomised Trials Including Multiple Births When Birth Size Is Informative. Paediatr Perinat Epidemiol. 2015;29(6):567–75.

28. Forbes AB, Akram M, Pilcher D, Cooper J, Bellomo R. Cluster randomised crossover trials with binary data and unbalanced cluster sizes: Application to studies of near-universal interventions in intensive care. Clin Trials. 2015;12(1):34–44.

29. Morgan KE, Forbes AB, Keogh RH, Jairath V, Kahan BC. Choosing appropriate analysis methods for cluster randomised cross-over trials with a binary outcome. Stat Med. 2016;36(2):318–33.

30. Barker D., D’Este C., Campbell M.J., McElduff P. Minimum number of clusters and comparison of analysis methods for cross sectional stepped wedge cluster randomised trials with binary outcomes: A simulation study. Trials. 2017;18(1):119.

31. Leyrat C., Morgan K.E., Leurent B., Kahan B.C. Cluster randomized trials with a small number of clusters: Which analyses should be used? Int J Epidemiol. 2018;47(1):321–31.

32. Borhan S, Mallick R, Pillay M, Kathard H, Thabane L. Sensitivity of methods for analyzing continuous outcome from stratified cluster randomized trials – an empirical comparison study. Contemp Clin Trials Commun. 2019;15:100405–100405.

33. Perin J, Preisser JS. Alternating logistic regressions with improved finite sample properties. Biometrics. 2016;73(2):696–705.

34. Bossoli D, Bottai M. Marginal quantile regression for dependent data with a working odds-ratio matrix. Biostatistics. 2018;19(4):529–45.

35. Prague M, Wang R, Stephens A, Tchetgen Tchetgen E, DeGruttola V, Tchetgen ET, et al. Accounting for interactions and complex inter-subject dependency in estimating treatment effect in cluster-randomized trials with missing outcomes. Biometrics. 2016;72(4):1066–77.

36. Lam KF, Ip D. REML and ML estimation for clustered grouped survival data. Stat Med. 2003;22(12):2025–34.

37. Peters TJ, Richards SH, Bankhead CR, Ades AE, Sterne JAC. Comparison of methods for analysing cluster randomized trials: An example involving a factorial design. Int J Epidemiol. 2003;32(5):840–6.

38. Kang W, Lee MS, Lee Y. HGLM versus conditional estimators for the analysis of clustered binary data. Stat Med. 2005;24(5):741–52.

39. Young ML, Preisser JS, Qaqish BF, Wolfson M. Comparison of subject-specific and population averaged models for count data from cluster-unit intervention trials. Stat Methods Med Res. 2007;16(2):167–84.

40. Olsen MK, DeLong ER, Oddone EZ, Bosworth HB. Strategies for analyzing multilevel cluster-randomized studies with binary outcomes collected at varying intervals of time. Stat Med. 2008;27(29):6055–71.

41. Sauzet O, Wright KC, Marston L, Brocklehurst P, Peacock JL. Modelling the hierarchical structure in datasets with very small clusters: A simulation study to explore the effect of the proportion of clusters when the outcome is continuous. Stat Med. 2013;32(8):1429–38.

42. Charvat H, Remontet L, Bossard N, Roche L, Dejardin O, Rachet B, et al. A multilevel excess hazard model to estimate net survival on hierarchical data allowing for non-linear and non-proportional effects of covariates. Stat Med. 2016;35(18):3066–84.

43. Pedroza C, Truong VTT. Estimating relative risks in multicenter studies with a small number of centers - which methods to use? A simulation study. Trials [Internet]. 2017;18(1). Available from: https://www.scopus.com/inward/record.uri?eid=2-s2.0-85032719571&doi=10.1186%2Fs13063-017-2248-1&partnerID=40&md5=71cc5945ce939d8f56555335b5973f41

44. Chen BE, Wang J. Joint modeling of binary response and survival for clustered data in clinical trials. Stat Med. 2019;(August):1–14.

45. Tawiah R, Yau KKW, McLachlan GJ, Chambers SK, Ng SK. Multilevel model with random effects for clustered survival data with multiple failure outcomes. Stat Med. 2019;38(6):1036–55.

46. Molas M, Lesaffre E. Hurdle models for multilevel zero-inflated data via h-likelihood. Stat Med. 2010;29(30):3294–310.

47. Borhan S, Kennedy C, Ioannidis G, Papaioannou A, Adachi J, Thabane L. An empirical comparison of methods for analyzing over-dispersed zero-inflated count data from stratified cluster randomized trials. Contemp Clin Trials Commun. 2020;17:100539–100539.

48. Christian NJ, Ha ID, Jeong JH. Hierarchical likelihood inference on clustered competing risks data. Stat Med. 2016;35(2):251–67.

49. Lu SE, Wang MC. Marginal analysis for clustered failure time data. Lifetime Data Anal. 2005;11(1):61–79.

50. Chen C.-M., Yu C.-Y. A two-stage estimation in the Clayton-Oakes model with marginal linear transformation models for multivariate failure time data. Lifetime Data Anal. 2012;18(1):94–115.

51. Cai J, Kim J. Nonparametric quantile estimation with correlated failure time data. Lifetime Data Anal. 2003;9(4):357–71.

52. Thompson SG, Warn DE, Turner RM. Bayesian methods for analysis of binary outcome data in cluster randomized trials on the absolute risk scale. Stat Med. 2004;23(3):389–410.

53. Müller P, Quintana FA, Rosner GL. Semiparametric Bayesian inference for multilevel repeated measurement data. Biometrics. 2007;63(1):280–9.

54. Ma J, Thabane L, Kaczorowski J, Chambers L, Dolovich L, Karwalajtys T, et al. Comparison of Bayesian and classical methods in the analysis of cluster randomized controlled trials with a binary outcome: the Community Hypertension Assessment Trial (CHAT). BMC Med Res Methodol. 2009;9(1):37–37.

55. Clark AB, Bachmann MO. Bayesian methods of analysis for cluster randomized trials with count outcome data. Stat Med. 2010;29(2):199–209.

56. Brown RL. Modeling impure clusters in a cluster randomized controlled trial. Res Nurs Health. 2013;36(2):216–23.

57. Ho M wai, Tu W, Ghosh P, Tiwari RC. Journal of the American Statistical Association A Nested Dirichlet Process Analysis of Cluster Randomized Trial Data With Application in Geriatric Care Assessment A Nested Dirichlet Process Analysis of Cluster Randomized Trial Data With Application in Ger. J Am Stat Assoc. 2013;(July 2014):37–41.

58. Li Z, Xu X, Shen J. Semiparametric Bayesian analysis of accelerated failure time models with cluster structures. Stat Med. 2017;36(25):3976–89.

59. Pan C, Cai B, Wang L. Multiple frailty model for clustered interval-censored data with frailty selection. Stat Methods Med Res. 2017;26(3):1308–22.

60. Balzer LB, Petersen ML, van der Laan MJ, Collaboration the S. Targeted estimation and inference for the sample average treatment effect in trials with and without pair-matching. Stat Med. 2016;35(21):3717–32.
