# Supplementary material for: Analysing cluster randomised controlled trials using GLMM, GEE1, GEE2, and QIF: results from four case studies
Source: BMC Med Res Methodol. 2023 Dec 13;23:293. doi: 10.1186/s12874-023-02107-z (PMC10717070; doi:10.1186/s12874-023-02107-z)
Supplement: Supplementary file 4 — Additional file 4. SAS syntax and R code for fitting the models on PoNDER trial data set only SAS syntax. [file 12874_2023_2107_MOESM4_ESM.docx]

**APPENDIX D**

# **SAS syntax and R code for fitting the models on PoNDER trial data set only**

**SAS syntax**

******************************************************************************;

* MODELING CONTINUOUS OUTCOME ;

* ;

******************************************************************************;

/*#####################GLMM-MLE MODELS############################;

/* Unadjusted/univariate model;

proc glimmix data = ponder method = quad(qpoints=10);

class group2(ref="0") clusteri;

model epds_6mo = group2/ dist=normal ddfm=bw cl;

random intercept / subject = clusteri s type=vc g;

output out=pon_cont0 resid=r0;

run;

/* Adjusted/multivariate model;

proc glimmix data=ponder method = quad(qpoints=10);

class group2(ref="0") clusteri alone history any_life;

model epds_6mo = group2 epds_6we alone history any_life/ dist=normal ddfm=bw cl;

random intercept / subject = clusteri s type=vc g;

output out=pon_cont1 pred=p resid=r1;

run;

/*#####################mGLM-QIF MODELS############################;

/* Calling the QIF macro to be used permanently throughout a session;

options mautosource sasautos = ("C:\Users\cmp17bco\Desktop\WP_1_ SAS\MACROS", sasautos);

%qif

/* Unadjusted/univariate model;

%qif(data=ponder,

yvar=epds_6mo,

xvar=group2,

id=clusteri, dist=normal, corr=exch,

print=y);

run;

/* Adjusted/multivariate model;

%qif(data=ponder,

yvar=epds_6mo,

xvar=group2 epds_6we alone history any_life,

id=clusteri, dist=normal, corr=exch,

print=y);

run;

******************************************************************************;

* MODELING BINARY OUTCOMES ;

* ;

******************************************************************************;

/*#####################GLMM-MLE MODELS############################;

/* Unadjusted/univariate model;

proc glimmix data=ponder method = quad(qpoints=10);

class group2(ref="0") clusteri;

model atrisk6m = group2 / dist=bin s ddfm=bw cl;

random intercept / subject = clusteri type= vc g;

run;

/* Adjusted/multivariate model;

proc glimmix data=ponder method = quad(qpoints=10);

class group2(ref="0") clusteri alone history any_life;

model atrisk6m = group2 epds_6we alone history any_life/ dist=bin s ddfm=bw cl;

random intercept / subject = clusteri type=vc g;

run;

/*#####################mGLM-QIF MODELS############################;

/* Unadjusted/univariate model;

%qif(data=ponder,

yvar=atrisk6m,

xvar=group2,

id=clusteri, dist=bin, corr=exch, descend=y,

print=y);

run;

/* Adjusted/multivariate model;

%qif(data=ponder,

yvar=atrisk6m,

xvar=group2 epds_6we alone history any_life ,

id=clusteri, dist=bin, corr=exch, descend=y,

print=y);

run;

***R codes***

###INSTALL REQUIRED PACKAGES##################

install.packages("lme4")

install.packages("geepack")

install.packages("qif")

###LOAD INSTALLED PACKAGES###################

library(lme4)

library(geepack)

library(qif)

###############################################################

################### MODELING CONTINUOUS OUTCOMES########

##############################################################

#######################################################################

####################mGLM-GEE1 MODELS##############################

#Unadjusted/Univariate model

UnAdjusted_GEE1_Con<-geeglm(epds_6mo~group2, family=gaussian, data=PONDER, id=clusteri, corstr = "exc")

Summary(UnAdjusted_GEE1_Con)

#Adjusted model/Multivariate model

# #Define the data set

PONDER_Adjusted_Con<-select(PONDER,epds_6mo,clusteri,group2,epds_6we,alone,history,any_life)

PONDER_Adjusted_Con<-na.omit(PONDER_Adjusted_Con)# Missing data must be handled for mGLM-GEE1 and mGLM-GEE2

Adjusted_GEE1_Con<-geeglm(epds_6mo~group2+epds_6we+ alone +history +any_life,family=gaussian,data= PONDER_Adjusted_Con,

id=clusteri, corstr = "exc")

summary(Adjusted_GEE1_Con)

##################################################################

#################mGLM-GEE2 MODELS#############################

#Unadjusted/univariate model

UnAdjusted_GEE2_Con<-geese(epds_6mo~group2,data=PONDER, id=clusteri, family=gaussian(link="identity"), corstr="exchangeable", cor.link = "fisherz", sca.link = "identity")

summary(UnAdjusted_GEE2_Con)

#Adjusted/Multivariate model

##Fitting the adjusted model with complete cases

Adjusted_GEE2_Con<-geese(epds_6mo~group2+epds_6we+alone+history+any_life, data= PONDER_Adjusted_Con, id=clusteri, family=gaussian(link="identity"), corstr="exchangeable",cor.link = "fisherz", sca.link = "identity")

summary(Adjusted_GEE2_Con)

#######################################################################

####################MODELING BINARY OUTCOMES#####################

#######################################################################

##To model binary outcomes, two changes were made to all the models of the continuous outcomes

## (1) The response variable is a binary variable (with only two possible outcomes e.g., 1 or 0).

## (2) The logit link function of the binomial family is used.

##A univariate example is given below, where the binary outcome is the “atrisk6m” variable

#######################################################################

####################mGLM_GEE1 MODELS##############################

######################################################################

#Unadjusted model

UnAdjusted_mGLM_GEE1<-geeglm(atrisk6m~group2,family=binomial(link = "logit"),data=PONDER,

id=clusteri,corstr = "exc")

summary(UnAdjusted_mGLM_GEE1)

# #Define the data set

PONDER_Adjusted_Bin<-select(PONDER, atrisk6m,clusteri,group2,epds_6we,alone,history,any_life)

PONDER_Adjusted_Bin<-na.omit(PONDER_Adjusted_Con)# Missing data must be handled for mGLM-GEE1 and mGLM-GEE2

#Adjusted model

Adjusted_mGLM_GEE1<-geeglm(atrisk6m~group2+epds_6we+

alone +history +any_life,family=binomial(link = "logit"),data= PONDER_Adjusted_Bin,id=clusteri,corstr = "exc")

summary(Adjusted_mGLM_GEE1)

#######################################################################

#################### mGLM_GEE2 MODELS ##############################

######################################################################

#Unadjusted Model

UnAdjusted_mGLM_GEE2_Bi<-geese(atrisk6m~group2,data=PONDER, id=clusteri, family=binomial(link="logit"),

corstr="exchangeable”, cor.link = "fisherz", sca.link = "identity")

summary(UnAdjusted_mGLM_GEE2_Bi)

##Adjusted Model

Adjusted_mGLM_GEE2_Bi<-geese(atrisk6m~group2+epds_6we+alone+history+any_life,data=PONDER_Adjusted_Bin,id=clusteri, family=binomial(link="logit"), corstr="exchangeable", cor.link = "fisherz", sca.link = "identity")

summary(Adjusted_mGLM_GEE2_Bi)
